# siRNA delivery using intelligent chitosan-capped mesoporous silica nanoparticles for overcoming multidrug resistance in malignant carcinoma cells

**DOI:** 10.1038/s41598-021-00085-0

**Published:** 2021-10-15

**Authors:** Razieh Heidari, Pegah Khosravian, Seyed Abbas Mirzaei, Fatemeh Elahian

**Affiliations:** 1 Department of Medical Biotechnology, School of Advanced Technologies, Shahrekord University of Medical Sciences, Shahrekord, Iran; 2 Medical Plants Research Center, Basic Health Sciences Institute, Shahrekord University of Medical Sciences, Shahrekord, Iran; 3 Cellular and Molecular Research Center, Basic Health Sciences Institute, Shahrekord University of Medical Sciences, Shahrekord, Iran

**Keywords:** Biotechnology, Cancer, Cell biology, Molecular biology

## Abstract

Although siRNA is a promising technology for cancer gene therapy, effective cytoplasmic delivery has remained a significant challenge. In this paper, a potent siRNA transfer system with active targeting moieties toward cancer cells and a high loading capacity is introduced to inhibit drug resistance. Mesoporous silica nanoparticles are of great potential for developing targeted gene delivery. Amino-modified MSNs (NH_2_-MSNs) were synthesized using a modified sol–gel method and characterized by FTIR, BET, TEM, SEM, X-ray diffraction, DLS, and ^1^H-NMR. MDR1-siRNA was loaded within NH_2_-MSNs, and the resulting negative surface was capped by functionalized chitosan as a protective layer. Targeting moieties such as TAT and folate were anchored to chitosan via PEG-spacers. The loading capacity of siRNA and the protective effect of chitosan for siRNA were determined by gel retardation assay. MTT assay, flow cytometry, real-time PCR, and western blot were performed to study the cytotoxicity, cellular uptake assay, targeting evaluation, and MDR1 knockdown efficiency. The synthesized NH_2_-MSNs had a particle size of ≈ 100 nm and pore size of ≈ 5 nm. siRNA was loaded into NH_2_-MSNs with a high loading capacity of 20% w/w. Chitosan coating on the surface of siRNA-NH_2_-MSNs significantly improved the siRNA protection against enzyme activity compared to naked siRNA-NH_2_-MSNs. MSNs and modified MSNs did not exhibit significant cytotoxicity at therapeutic concentrations in the EPG85.257-RDB and HeLa-RDB lines. The folate-conjugated nanoparticles showed a cellular uptake of around two times higher in folate receptor-rich HeLa-RDB than EPG85.257-RDB cells. The chitosan-coated siRNA-NH2-MSNs produced decreased MDR1 transcript and protein levels in HeLa-RDB by 0.20 and 0.48-fold, respectively. The results demonstrated that functionalized chitosan-coated siRNA-MSNs could be a promising carrier for targeted cancer therapy. Folate-targeted nanoparticles were specifically harvested by folate receptor-rich HeLa-RDB and produced a chemosensitized phenotype of the multidrug-resistant cancer cells.

## Introduction

Since their discovery in 1998, RNA interferences (RNAi) have attracted increasing attention in medical research due to their high suppressing potential against the expression of disease-promoting genes^1,2^. A gene knockdown in the RNAi pathway is initiated by transferring a chemically synthesized small interfering RNA (siRNA) containing 19–23 base pairs to the cytoplasm. While siRNAs provide potential opportunities in the medical healthcare system, their intrinsic properties, such as polyanionic character, high molecular weight, short half-life, fast enzymatic degradation in biological systems, and low cellular uptake, restrict the therapeutic use of such nucleic acid-based drugs^3,4^.

Recently, mesoporous silica nanoparticles (MSNs) have gained popularity in research as efficient intracellular carriers for genes and poorly soluble chemotherapeutics due to their cellular safety, excellent biocompatibility, controlled porous structure, high surface area, and unique conjugation properties with targeting moieties due to the abundant silanol groups on the surface^5,6^. Additionally, MSNs are used as controlled release systems by adding intelligent gatekeeper molecules. MSN's pore capping with gatekeeper molecules prevents the cargo from leaking before delivery to the target site, and drug release can occur only in response to a specific stimulus^7–10^. Passive targeting is another advantage of using MSN for cancer gene therapy, as 20–200 nm nanoparticles can passively accumulate at higher concentrations in tumor sites than in normal tissues, owing to the tumor vessels' enhanced permeability and retention (EPR) effect^11,12^. The EPR effect alone is insufficient to ensure effective nanoparticle uptake by cells. As a result, active targeting is frequently used to minimize off-target effects and maximize cell-specific delivery.

During active targeting, nanoparticles' surface can be decorated with protein, peptide, aptamer, sugar, vitamin, or antibody against surface antigens or unique receptors^12^. Herceptin, EGFs, VEGF, RGD, transferrin, mannose, hyaluronic acid, and folate are common ligand examples widely used to anchor the MSNs for cancer cells^13,14^. Folate mainly involves DNA synthesis and repair, DNA methylation, and cellular function, respectively. Alpha folate receptor (FRα), also known as folate binding protein (FBP), is a high-affinity membrane folate receptor with a low expression level in healthy tissues; however, it is up to 300 times overexpressed in epithelial cancers such as ovarian, breast, and lung^15^. On the other hand, favorable properties including safety, cost-effectiveness, non-immunogenicity, high stability, simple conjugation with various molecules, and binding affinity to the folate receptor (FR) after conjugation highlight the folate-targeting system as a considerable intelligent drug delivery system.

In this study, siMDR1 was used to break down the multiple drug-resistant protein-1 (MDR1) or P-glycoprotein (P-gp) in multidrug-resistant lines to induce chemosensitized phenotype. P-glycoprotein plays an essential role in churning out xenobiotics from normal cells, but its overexpression in cancer tissues produces a drug resistance phenotype due to chemotherapeutic drug efflux. As a result of the preceding, novel positively charged mesoporous silica nanoparticles for siMDR1 adsorption were synthesized. Then, chitosan was used to coat siMDR1 as a protective layer, as physical adsorption in gene delivery systems has been disputed due to RNA degradation by nucleases in physiological fluids. Poor solubility at physiological pH is a significant drawback of chitosan; thus, polyethylene glycol (PEG) was used to increase solubility and conjugate TAT plus folate. Functionalized chitosan was used for the coating of the siRNA-loaded nanoparticles^10,16,17^. Furthermore, a cellular model for such malignancy was produced by gene cloning to measure the delivery system's effectiveness. Conjugation of chitosan with folate and a cell-penetrating peptide such as TAT is regarded as an intelligent strategy for increasing active cellular uptake and facilitating cargo nanomolecules' endosomal escape.

## Results

### Synthesis, characterization, and loading capacity of MSN-NH_2_

**Figure 1:**
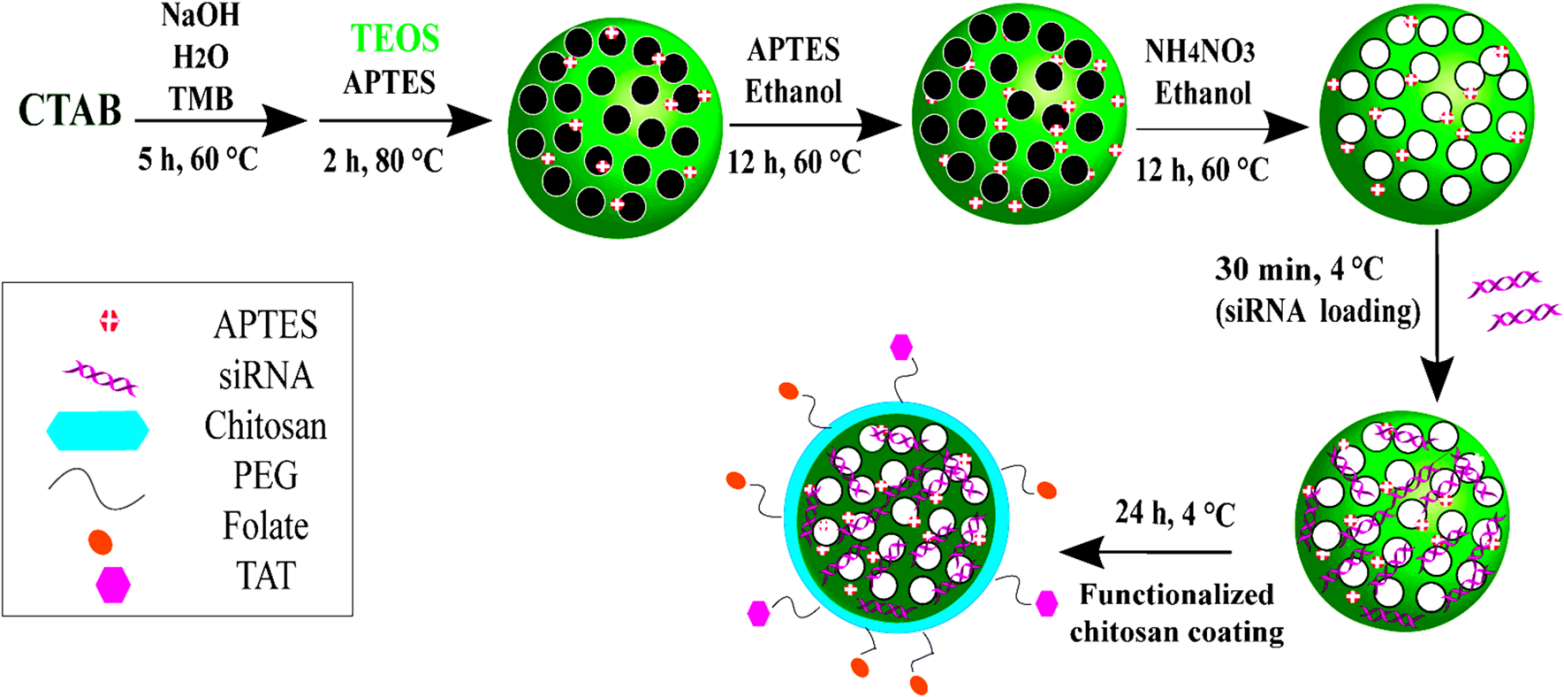
Schematic illustration of synthesis steps and mesoporous silica nanoparticles (MSNs) functionalization to obtain NH_2_-MSN, NH_2_-MSN-siRNA, and NH_2_-MSN-siRNA-chitosan functionalized with PEG-folate and PEG-TAT. *APTES* 3-Amino propyltriethoxysilane, *CTAB* cetyltrimethylammonium bromide, *PEG* polyethylene glycol, *TAT* trans-activator of transcription, *TMB* 1,3,5-triisopropylbenzene, *TEOS* tetraethyl orthosilicate.

**Figure 2:**
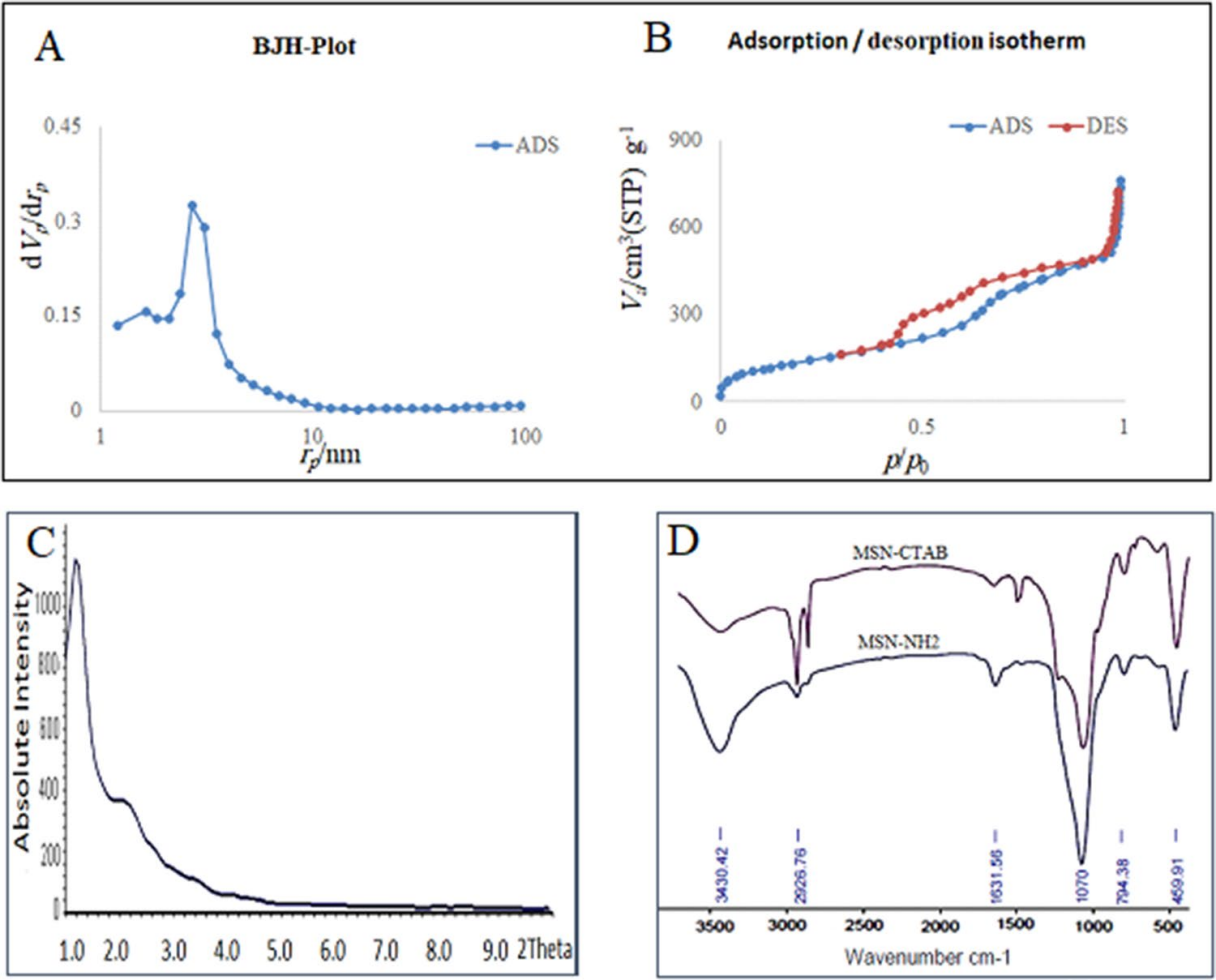
The distribution of pore sizes and the isotherms of nitrogen adsorption/desorption in MSN-NH_2_ (**A**,**B**). MSN-NH_2_XRD patterns revealed the locations of the peaks associated with the mesoporous silica structure (**C**). The FTIR spectra obtained before and after CTAB extraction from MSNs confirmed the successful elimination of CTAB and the addition of the aminopropyl moiety (**D**).

Figure 1 illustrates a schematic representation for the synthesis and application steps. Physical parameters of MSNs, including the surface area, pore pattern, and pore volume, were determined through the BET nitrogen adsorption/desorption method after CTAB removal. The pore diameter was calculated as 5 nm for NH_2_-MSNs according to the BJH method, and the BET surface area of MSNs was 512 m^2^/g (Fig. 2A,2B). The XRD pattern of NH_2_-MSNs revealed the retention of the 2D hexagonal shape of nanoparticles with a p6 mm symmetry (Fig. 2C).

**Figure 3:**
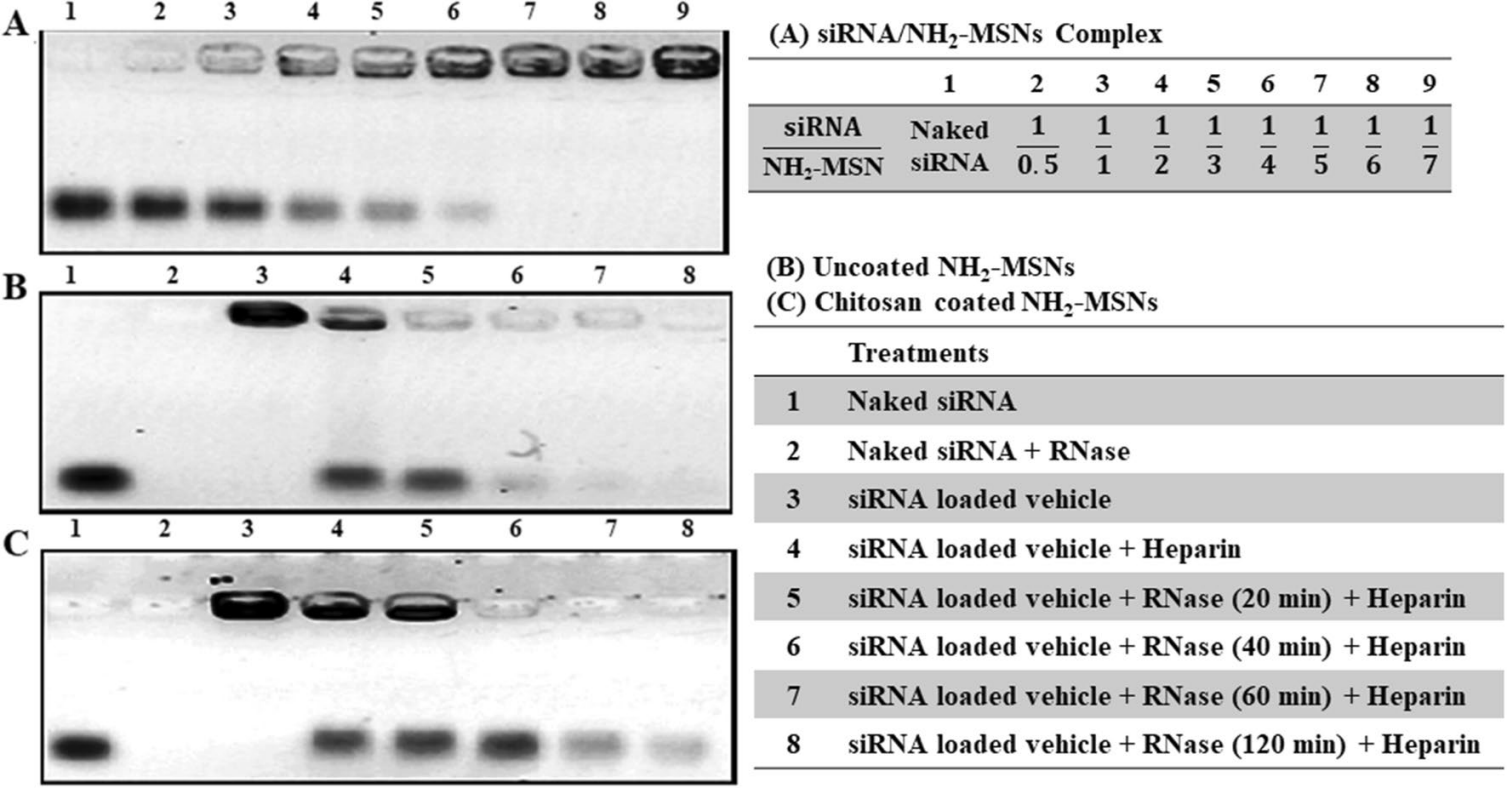
Electrophoretic mobility pattern of siRNA/MSN complexes from left to right: naked siRNA, 1:0.5, 1:1, 1:2, 1:3, 1:4, 1:5, 1:6, and 1:7 on agarose gel (**A**). RNase protection capacity for siRNA-loaded NH_2_-MSNs (**B**), and siRNA-loaded NH_2_-MSNs coated with chitosan (**C**). Wells from left to right: naked siRNA, naked siRNA treated with RNase, siRNA-loaded NH_2_-MSNs, heparin treated NH_2_-MSNs in the absence of the RNase, siRNA liberated from NH_2_-MSNs using heparin in the presence of the RNase for 20 min, for 40 min, for 60 min, and finally 120 min. Gel analysis revealed that MSNs coated with chitosan might provide greater protection for the loaded siRNA than MSNs not coated with chitosan. UVIDoc software version 12.4 was used to crop and color invert the images. The supplementary dataset contains original images.

The surfactant molecule (CTAB) was removed with NH_4_NO_3_ solution to form empty porous nanochannels in the as-prepared NH_2_-MSN. FTIR verified the success of CTAB surfactant extraction from the pores by eliminating the characteristic CTAB peaks in 1484 cm^−1^, 2890 cm^−1^, and 2919 cm^−1^ which indicated the methylene chains. The presence of positive charges on the inner and outer surface of MSN was confirmed via the new band allocated to the N–H bending vibration at ~ 1500 cm^−1^ (Fig. 2D). The gel retardation assay revealed that the siRNA migration entirely stopped when the weight ratio of siRNA/MSN was 1:5 in electrophoresis and a loading capacity of approximately 20% w/w (Fig. 3A). The siRNA loading in MSN-NH_2_ was further confirmed by shifting the zeta potential value from positive to negative after siRNA loading (Table 1).

**Table 1:** Zeta-potential of nanoparticles. *CS* Chitosan, *PEG* polyethylene glycol, *TAT* trans-activator of transcription.

Nanoparticle	Zeta potential (mV)
MSN	− 10.8 ± 1.5
NH_2_-MSN	16.2 ± 1.1
NH_2_-MSN-siRNA	− 15.2 ± 1.6
NH_2_-MSN-siRNA-CS	18.5 ± 2.2
NH_2_-MSN-siRNA-CS-PEG	− 8.4 ± 0.6
NH_2_-MSN-siRNA-CS-PEG-Folate	9.0 ± .1.5
NH_2_-MSN-siRNA-CS-PEG-TAT	9.8 ± 0.9
NH_2_-MSN-siRNA-CS-PEG TAT/folate	1.7 ± 0.6

### Targeted chitosan coats and MSN-siRNA capping

**Figure 4:**
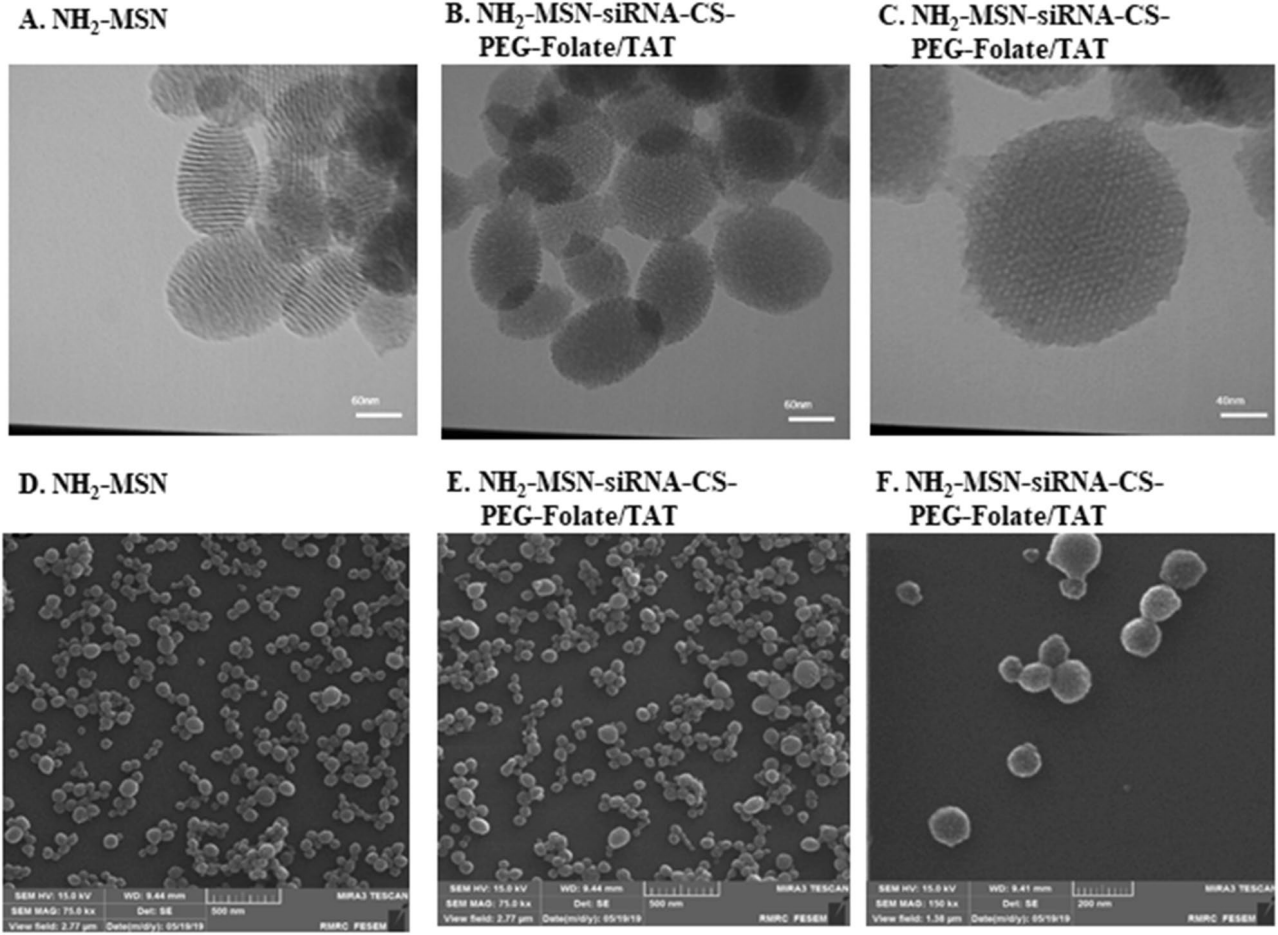
TEM and SEM images of the nanoparticles. NH_2_-MSNs at TEM and SEM monographs represent around 70 nm (**A**,**D**). NH_2_-MSN-siRNA particles coated with chitosan-PEG(3000)-folate/TAT were presented on TEM (**B**,**C**) and also on SEM images (**E**,**F**) with different zooming factors. The images represent homogenous particles around 100 nm.

Chitosan was conjugated with the sulfhydryl group of SH-folate and SH-cysteine-TAT using a PEG spacer to improve tumor specificity and MSN uptake. The SH-folate for such reaction was obtained using 2-Mercaptoacetic acid with an average synthesis yield of 70.7 ± 5.5%. A complete reaction procedure is depicted in Supplementary Fig. S1. TLC was used to confirm the reaction between Cys-TAT and MAL-PEG(3000)-NHS; then, using Ellman's assay, the conjugation efficiency of SH-folate and Cys-TAT to the maleimide group of MAL-PEG(3000)-NHS was determined to be 86.5% and 70.2%, respectively (Supplementary Fig. S2). The average synthesis yields for those reactions were 82.2% and 65.4%, respectively. The NHS moieties in folate-PEG(3000)-NHS or TAT-PEG(3000)-NHS were substituted with the primary amine of chitosan to form folate-PEG(3000)-chitosan, TAT-PEG(3000)-chitosan, and double folate/TAT modified chitosan, yielding 96.3%, 95.5%, and 90.3% modified chitosan, respectively (Supplementary Fig. S3). ^1^H-NMR spectra of chitosan displayed characteristic peaks around 3.1 ppm corresponding to the monosaccharide residue protons. Sharp peaks confirmed the successful synthesis of chitosan-PEG(3000) conjugates at 3.1–4.0 ppm representing ethylene repeats in PEG. The substitution degree of PEG(3000) residues on chitosan was 1.7% using ^1^H-NMR (Supplementary Fig. S4). After mixing, chitosan reacted to the NH_2_-MSN-siRNA through electrostatic bonds. Zeta potential values of MSN, NH_2_-MSN, NH_2_-MSN-siRNA, NH_2_-MSN-siRNA-chitosan, NH_2_-MSN-siRNA-chitosan-PEG(3000)-folate, NH_2_-MSN-siRNA-chitosan-PEG(3000)-TAT, and NH_2_-MSN-siRNA-chitosan-PEG(3000)-folate/TAT are summarized in the Table 1. Zeta-potential values of NH_2_-MSN structures were completely affected by the modifications. For example, the z-potential of MSN was − 10 mV due to silicon hydroxyl groups, whereas the z-potential of MSN-NH_2_ nanoparticles was + 16 mV due to amine groups (Table 1). NH_2_-MSN-siRNA coating with double folate/TAT modified chitosan was also confirmed on TEM images through rough surfaces and the loss of mesoporous structures of the nanoparticles (Fig. 4). The particle sizes of NH_2_-MSN and coated NH_2_-MSN-siRNA with double folate/TAT modified chitosan were determined to be 70 ± 12 nm and 81 ± 10 nm, respectively, using SEM images (Fig. 4). Using a DLS, the sizes of NH_2_-MSN and double folate/TAT modified chitosan-coated NH_2_-MSN-siRNA were determined to be approximately 80 and 110 nm, respectively. The larger nanoparticles determined by DLS were caused by the hydrodynamic layer surrounding the particles in the solution (Supplementary Fig. S5). Overall data obtained from the coated and uncoated NH_2_-MSN through TEM, SEM, and DLS confirmed the successful deposition of a functionalized chitosan on the nanoparticle surface. UV-spectroscopy at 260 nm was used to monitor siRNA leakage from nanoparticles for 4 days in PBS. The formulation exhibited no evidence of siRNA leakage and exhibited high stability over 4 days at 4 °C (Fig. 3).

### Chitosan protection capacity on siRNA

The protective effect of chitosan on siRNA-MSNs was examined through agarose gel electrophoresis. Enzymatic cleavage of siRNA in the physiological fluid is a critical obstacle in gene therapy. Thus, NH_2_-MSN-siRNA and NH_2_-MSN-siRNA-chitosan were treated with RNase-A to evaluate siRNA protection capability. Naked siRNA was totally digested by RNase-A incubation before gel electrophoresis (Fig. 3B,C; lane 2). NH_2_-MSN-siRNA and NH_2_-MSN-siRNA-chitosan remained in the gel wells due to the complete binding of siRNA to NH_2_-MSN (Fig. 3B,C; lane 3). Lane 4 demonstrated the release of siRNA from nanoparticles in the presence of heparin (Fig. 3B,C). Heparin is a negatively charged biomolecule that is frequently used in competition with siRNA to bind cationic vectors. The dissociation of siRNA from siRNA/MSN complexes is a frequently used technique in studies to determine siRNA release. The bound siRNA in the NH_2_-MSN-siRNA-chitosan sample remained intact following RNase-A treatment (Fig. 3C; lane 5), but the intensity of the bound siRNA in the NH_2_-MSN-siRNA complex was significantly reduced following heparin exposure (Fig. 3B; lane 5). By increasing the enzyme treatment time, the siRNA bands in chitosan-coated nanoparticles (Fig. 3C, lanes 6, 7, and 8) remained identifiable compared to the naked siRNA in NH_2_-MSN-siRNA (Fig. 3B; lanes 6, 7, and 8).

### MDR1 expressing cell models development and characterization

HeLa cells were transfected with lentiviral particles carrying the MDR1 gene (Supplementary Fig. S6). The best transfection results were obtained at MOI = 5, and recombinant cells were selected during 5 days using the least toxic dose of hygromycin-B for HeLa cells (200 µg/ml, Supplementary Fig. S7). A stable single-cell overexpressing 58.4-folds of MDR1 was selected and labeled as HeLa-RDB. This clone expressed 297.1-fold more folate receptors than EPG85.257-RDB (p < 0.001, Supplementary Fig. S8). Doubling times were calculated at 34.5, 22.6, 64.8, and 69.3 h for EPG85.257, EPG85.257-RDB, HeLa, and HeLa-RDB cells, respectively (Supplementary Fig. S9).

### Biocompatibility and nanoparticle cytotoxic kinetics

**Figure 5:**
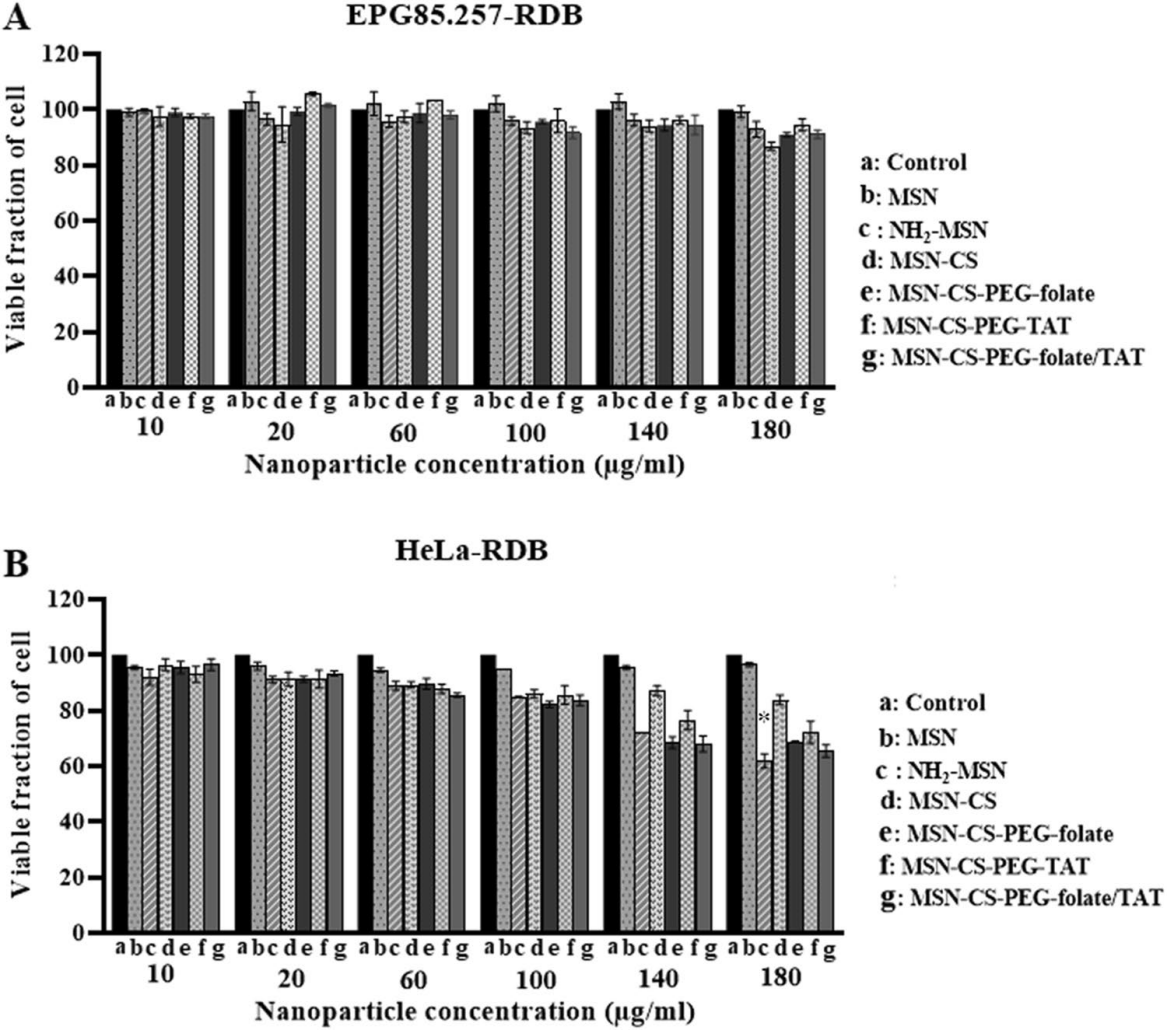
Cytotoxicity evaluation of the nanostructures. EPG85.257-RDB and HeLa-RDB viability after 72 h incubation with nanoparticle-free media (a), MSN (b), NH_2_-MSN(c), MSN-CS(d), MSN-CS-PEG-folate (e), MSN-CS-PEG-TAT (f), and MSN-CS-PEG-folate/TAT (g). Nanoparticles were found to be biocompatible, and the treatment concentration should not exceed 100 μg/ml. Data showed that nanoparticles were biocompatible, and the treatment concentration should not be exceeded 100 μg/ml. *p < 0.05 denote significant differences between the control and treatment samples according to the one-way ANOVA (non-parametric Kruskal–Wallis analyses). *CS* Chitosan, *PEG* polyethylene glycol, *TAT* trans-activator of transcription.

The MTT assay was used to determine cellular toxicity following treatment with MSNs structures. Figure 5 shows that MSNs, NH_2-_MSN, and any functional coated MSNs had insignificant toxicity on EPG85.257-RDB (p > 0.05). However, some coated particles influenced the viability of HeLa-RDB cells at very high concentrations. Microscopic images of the cells after 72 h nanoparticle treatments confirmed no toxic influences, and no discrepancy was found between microscopy images of the cells with or without nanoparticles treatment (Supplementary Fig. S10). On the other hand, daunorubicin toxicity was significantly increased in siRNA delivered cells. Increased exposure to folate receptors rendered HeLa-RDB more susceptible to daunorubicin toxicity, owing to the final NH_2_-MSN-siRNA-chitosan-PEG(3000)-folate/TAT nanoparticles' greater silencing of the MDR1 (Table 2).

**Table 2:** IC_50_ values of daunorubicin in the presence of 5 µg/ml of each nanoparticle on EPG85.257-RDB and HeLa-RDB cells. Data were expressed as mean ± SD. Stars (*), (**), and (***) represent p < 0.05, p < 0.01, and p < 0.001, respectively. *CS* Chitosan, *PEG* polyethylene glycol, *TAT* trans-activator of transcription.

Treatments	Daunorubicin IC_50_ (nM) on EPG85.257-RDB cells	Daunorubicin IC_50_ (nM) on HeLa-RDB cells
No nanoparticle treatment	6412.1 ± 572	80.14 ± 6.2
NH_2_-MSN-siRNA	1809.8 ± 304	55.12 ± 3.6
NH_2_-MSN-siRNA-CS	784.7 ± 51	37.40 ± 6.1
NH_2_-MSN-siRNA-CS-PEG-folate	1138.1 ± 149	22.31 ± 3.6**
NH_2_-MSN-siRNA-CS-PEG-TAT	440.2 ± 34**	26.15 ± 6.1*
NH_2_-MSN-siRNA-CS-PEG folate/TAT	245.4 ± 55***	13.88 ± 3.0***

### Cellular uptake and MDR1 gene silencing

**Figure 6:**
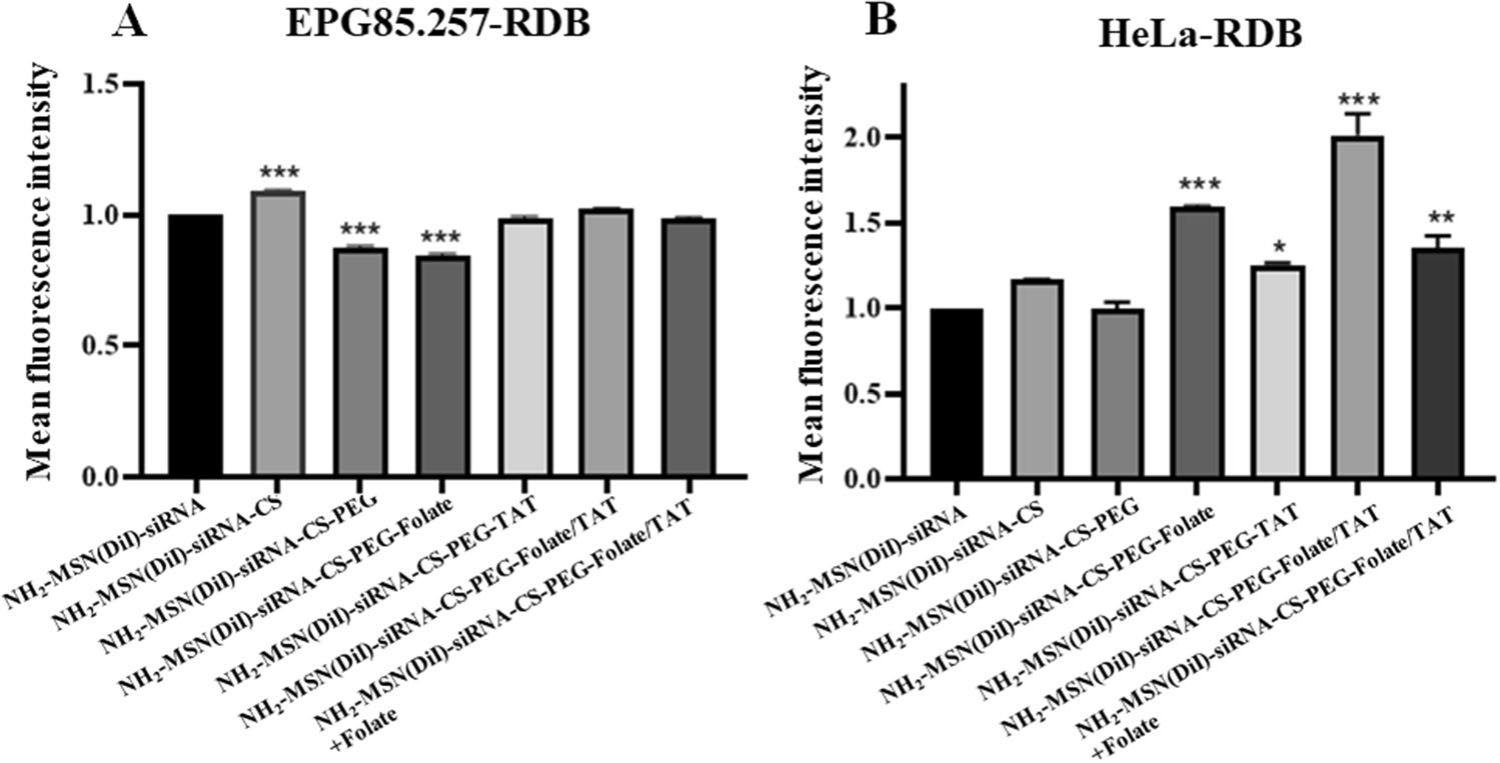
Assessment of the cell uptake efficiencies of various nanoparticles. Flow cytometry analysis in EPG85.257-RDB and HeLa-RDB indicated the successful intracellular uptake of functionalized chitosan-coated NH_2_-MSNs in HeLa-RDB cells. Symbols (*), (**) and (***) denote significant differences between the control (naked NH_2_-MSNs) and coated samples by p < 0.05, p < 0.01 and p < 0.001. *MFI* Mean fluorescence intensity, *CS* chitosan, *PEG* polyethylene glycol, *TAT* trans-activator of transcription.

Flow cytometry was used to assess the internalization ability of the functionalized chitosan-coated nanoparticles as an active delivery system in HeLa-RDB (high-expression folate receptor) and a passive delivery system in EPG85.257-RDB cells (low-expression folate receptor). Based on the flow cytometry results, the fluorescent intensities of TAT/folate modified NH_2_-MSNs were more potent in the HeLa-RDB line compared to the control (uncoated NH_2_-MSNs) (p < 0.001), indicating that the folate-linked nanoparticles could effectively target folate receptors on HeLa-RDB cells (Fig. 6 and Supplementary Fig. S13). The results indicate that TAT-conjugated nanoparticles significantly increase cellular uptake of nanoparticles in both cell lines when the control sample is considered to be PEG-chitosan-NH_2_-MSN treated cells. This phenomenon shows a positive role for the TAT in the interaction with the cell membrane and cell entry. PEGylation of chitosan resulted in decreased cellular uptake and transfection efficiency for various reasons, including inhibition of cellular uptake (repulsion from PEG) and inefficient endosomal escape. Targeting moieties (TAT or folate) were covalently bonded to the free ends of PEG to improve cellular uptake by allowing them to recognize the expressed receptors over the cell membrane, specifically. Cells' uptake of PEGylated nanoparticles is significantly increased due to a specific ligand-receptor interaction.

**Figure 7:**
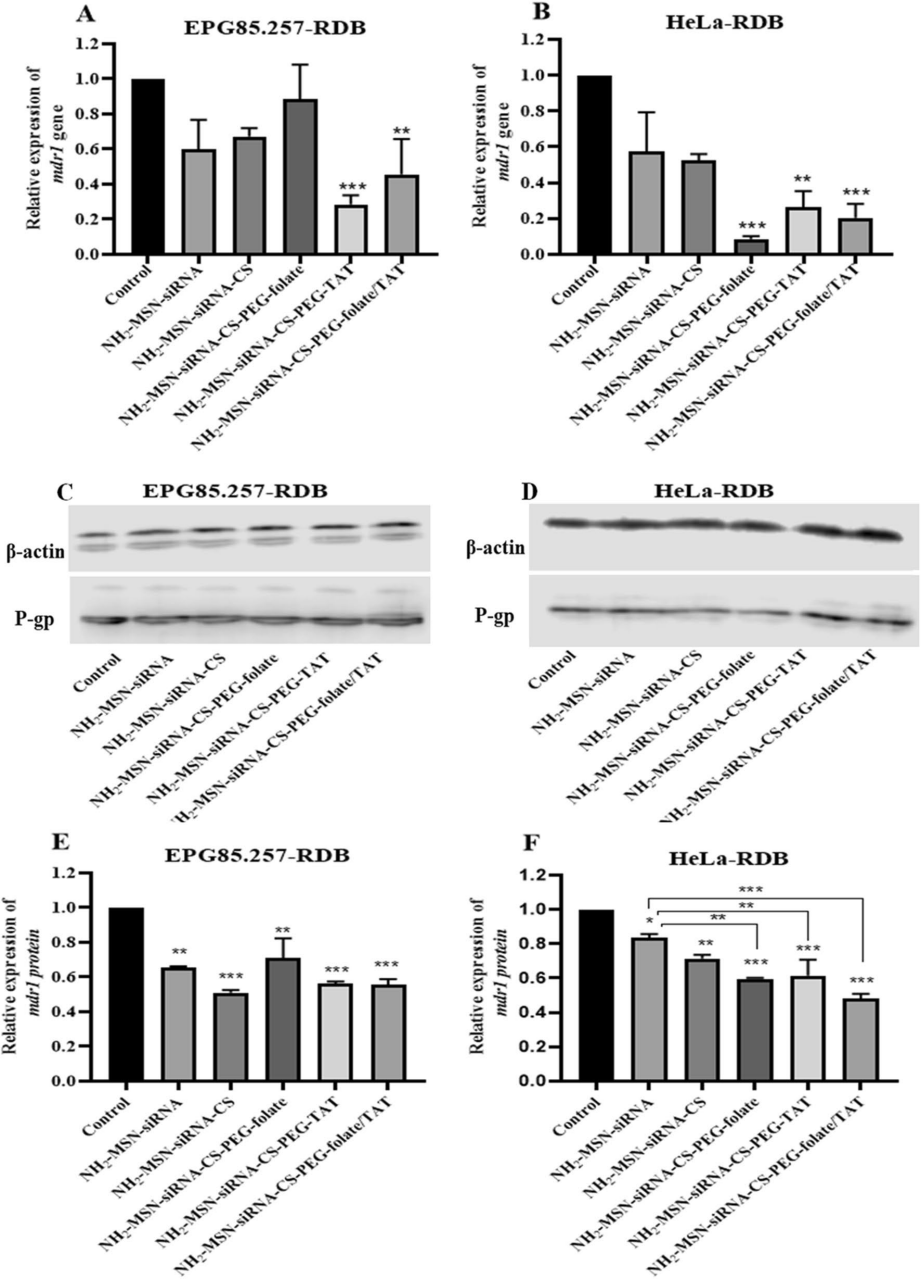
Molecular effectiveness of siMDR1 delivery by MSNs. After 48 h of exposure to various nanoparticle structures, the MDR1 gene was knocked down using real-time PCR (**A**,**B**) and western immunoblotting (**C**,**D**). The functionality of the siMDR1 nanocarriers against the cell lines revealed a significant downregulation of MDR1 transcript and protein in HeLa-RDB cells in the presence of NH_2_-MSN-chitosan-PEG-folate/TAT. The western band intensities of MDR1 were quantified and normalized to β-actin using Image Studio software (**E**,**F**). *p < 0.05, **p < 0.01, and ***p < 0.001 denote significant differences between the negative control (NC-siRNA loaded NH_2_-MSN-chitosan-PEG-folate/TAT construct) and treatment samples. *CS* Chitosan, *PEG* polyethylene glycol, *TAT* trans-activator of transcription. Original blot pictures are included in the supplementary dataset**.**

siRNA delivery through mesoporous silica nanoparticles led to a significant reduction of MDR1 transcripts and proteins after 48 h, where TAT/folate modified chitosan was the strongest structure, and HeLa-RDB was the most influenced line (p < 0.001; Fig. 7, Supplementary Table S1, and Supplementary Fig. S14). Real-time PCR analyses demonstrated that siMDR1-loaded NH_2_-MSNs-chitosan-PEG-TAT significantly reduces MDR1 transcription in both cell lines due to TAT's high cell membrane penetration and proton sponge properties. Gene silencing via siMDR1-loaded NH_2_-MSNs-chitosan-PEG-TAT/folate construct was great on HeLa-RDB due to the corresponding folate receptor on this cell and active transportation. In the western analyses, decreases in protein expression were observed in all samples, but the significance of these reductions varied. Hela-RDB treated cells with NH_2_-MSN-siRNA-CS-PEG-folate/TAT showed the greatest decrease (***p < 0.001). Western blotting results are almost consistent with the real-time PCR outputs.

## Discussion

The current study's objective was to develop MSN-based nanocarriers for tumor-specific gene therapy and induce a chemosensitized phenotype in multidrug-resistant carcinoma cells. MSNs have been employed as siRNA delivery vehicles due to their favorable properties, including a network of hollow cavities, high loading capacity, large surface areas (ca. 1000 m^2^/g), high pore volumes (ca. 1 cm^3^/g), biocompatibility, and facile surface modification^18,19^. The fabrication of MSNs is cost-effective and straightforward. However, some limitations remain in the successful translation of this system to the bedside. Recently, several promising results demonstrating the remarkable potential of MSNs for drug delivery in cancer therapy have been gathered^20,21^.

NH_2_-MSNs were designed and synthesized using a base-catalyzed sol–gel method for siRNA loading. TEM images showed successful syntheses of NH_2_-MSNs and represented the bright and dark areas corresponding to the pores and the silica walls of NH_2_-MSNs, respectively. TEM also confirmed organized nanochannels in a hexagonal structure. The particle size of NH_2_-MSNs was approximately 70 ± 12 nm, as determined by the ImageJ software in scanning electron microscopy (SEM) images, consistent with the TEM result (Fig. 4). The polydispersity index (PDI) value of 0.5 for NH_2_-MSNs indicated that the nanoparticles were approximately monodisperse; consistent with electron microscopy images. The BET isotherm curve obtained from the NH_2_-MSNconfirmed the typical type-IV isotherm curve with a hysteresis loop and a noticeable step between 0.3 and 0.4 p/p0, indicating the presence of mesoporous structures^22^.

The hexagonal arrays of pores in the NH_2_-MSNs were confirmed by XRD and BET results. The XRD pattern (Fig. 2C) indicated a slight shift in the d_00_ value, which could result from the addition of TMB as a micelle pore expander and the covalent binding of amine groups in the pores or on the walls of MSNs^23^. MSNs were aminated with APTES to facilitate siRNA loading, as tiny aminopropyl grafts on the mesopores enhance biocompatibility and adsorption capacity. The disappearance of the 960 cm^−1^ bands on FTIR spectra demonstrated that MSN surfaces were adequately aminated (Fig. 2D). Additionally, several peaks can be attributed to the C–H stretching vibrations of the aliphatic chain at 2900 cm^−1^, implying that APTES is anchored to MSN surfaces^22,24,25^.

The ability of NH_2_-MSN to interact with siRNA was confirmed using gel assay (Fig. 3A), indicating that the loading capacity of siRNA is approximately 20% w/w, which is a high loading record for a modified medium-sized mesopore of approximately 5 nm (Fig. 2A). Such pore size is freely available for a common siRNA around 2 nm^26^. While some researchers demonstrated that large-pore MSNs could adsorb more significant amounts of siRNA within the pores, they are susceptible to RNase diffusion and siRNA digestion due to the RNase's small size. Furthermore, the assembled gatekeeper or protective shield on the large-pore MSNs needs complex multistep preparation processes^27,28^. On the other hand, the positive charge and porous nature of NH_2_-MSN enable siRNA adsorption on the outer surface and loading within the pores after a specific orientation is adopted. Physically attached siRNAs on the nanoparticle surfaces may be easily degraded by an endonuclease, and prolonged incubation time may allow siRNAs to be released from pores before delivery to the target site. The NH_2_-MSN-siRNA surface was coated with chitosan to protect siRNA from enzymatic cleavage, and gel assay analyses revealed that chitosan coating could protect siRNA from enzymatic cleavage (Fig. 3B,C). The negative charges of the siRNA are needed for efficient electrostatic interactions of chitosan. In addition, the protective effect of chitosan coats facilitated the surface modification of NH_2_-MSN with multifunctional ligands. PEGylation of chitosan was used in this study to increase its water solubility, prevent the reticuloendothelial system from detecting the chitosan-based nanocarriers, and prolong their circulation time^29^. Besides, the medium-sized PEG created a suitable linker for the folate and TAT moieties (Supplementary Fig. S3).

Conjugation of folate-PEG or TAT-PEG to chitosan covered the positive charge in modified chitosan-coated NH_2_-MSN-siRNA, which was in agreement with zeta-potential measurement results (Table 1). The zeta potential value confirmed the presence of positive charges on both inner and outer surfaces of NH_2_-MSN. The zeta potential of MSNs was − 10.8 mV due to the silicon hydroxyl groups, whereas the zeta potential of NH_2_-MSN nanoparticles was + 16 mV due to the amine groups. The electrostatic interaction of negatively charged siRNA with cationic NH_2_-MSN groups resulted in a shift in the zeta potential value from positive to negative. Then, coating the NH_2_-MSN-siRNA with chitosan increased the zeta-potential values from − 15 to + 18, due to the primary amine groups in chitosan. The zeta-potential values of chitosan-PEG-coated NH_2_-MSN-siRNA were negative due to the presence of large hydroxyl groups on PEG(3000) and balanced the chitosan's positive charges^30,31^. Modification of chitosan with PEG(3000)-folate or PEG(3000)-TAT resulted in positive zeta-potential values (around 9 mV), confirming that the targeting moieties (ligands) are covalently bonded to the PEG free ends. The presence of folate or TAT can mask the negatively charged hydroxyl groups on PEG(3000), increasing zeta-potential values. The zeta-potential of NH_2_-MSN-siRNA-chitosan-PEG(3000)-folate/TAT was 1.7 mV due to the presence of more PEG(3000) molecules on this coating, confirming the successful grating process^31–33^.

The final nanoparticle size was around 100 nm, which was ideal for EPR-induced tumor tissue accumulation. Although MSNs have been widely reported as safe and biocompatible for intracellular delivery, several studies have found that MSNs are toxic above 100 µg/ml when APTES is incorporated^34^. Others revealed that APTES-modified nanoparticles were not toxic to cells even at higher concentrations^35^. In this study, the presence of APTES or functionalized chitosan on the MSN surface did not affect cell viability in EPG85.257-RDB or HeLa-RDB cells in the microscopic view (Supplementary Fig. S10); however, MTT results showed reduced viability of HeLa-RDB cells after treatment with NH_2_-MSN or functionalized chitosan-coated NH_2_-MSNs (Fig. 5). There is evidence in a report that treatment of HeLa cells with functionalized MSNs induces MTT exocytosis and decreases intracellular formazan crystals, but this is not associated with decreased cell viability^36^. PEG and chitosan are biodegradable polymers in their entirety, and their presence on the MSN structure has no effect on cellular viability at therapeutic concentrations^16^.

Final constructs were used as targeting agents for HeLa-RDB cells expressing high levels of folate receptors and EPG85.257-RDB cells expressing low levels of folate receptors. HeLa-RDB cells internalized DiI-NH_2_-MSN-chitosan-PEG-folate/TAT at a much higher rate than DiI-MSN, indicating that folate conjugation enhanced HeLa-RDB cell uptake. In contrast, EPG85.257-RDB cells inefficiently internalized DiI-NH_2_-MSN-chitosan-PEG-folate/TAT due to low folate expression. Moreover, the cellular internalization of DiI-NH_2_-MSN-chitosan-PEG-folate/TAT by HeLa-RDB cells was blocked in the presence of excess free folates. Simultaneously, this structure was not taken up by EPG85.257-RDB cells, indicating that the folate receptor on HeLa-RDB cells promotes endocytosis via folate ligands on the corresponding nanostructures (Fig. 6). There are numerous studies on the benefits of folate receptors in cancer targeting^37–40^. This paper demonstrated that conjugating folate with a nanostructure increase targeted therapy effectiveness because it induces tumor cells to uptake nanoparticles, selectively. On the other hand, pegylation decreased chitosan charges and nanoparticle uptake, consistent with the previous research^41^. TAT-modified nanocarriers accumulated a higher degree of NH_2_-MSNs within the cells, agreeing with the flow cytometry data. The proton sponge effect of chitosan and TAT promotes endosomal escape and release of the encapsulated cargo^42^. This outcome confirmed the functionality of the nanocarrier in the targeted delivery of siMDR1. Even though the NH_2_-MSN-siRNA and NH_2_-MSN-siRNA-chitosan nanoparticles decreased the MDR1 transcript and protein in both cells, more silencing results were observed in HeLa-RDB cells in the presence of final constructs (Fig. 7). Targeting NH_2_-MSN-siRNA with folic acid and TAT (NH_2_-MSN-siRNA-CS-PEG-TAT/Folate) improved the siRNA delivery in folate receptor-rich HeLa-RDB cells in comparison with NH_2_-MSN-siRNA due to effective uptake of folate-targeted nanoparticles. EPG85.257-RDB gene silencing using NH_2_-MSN-siRNA-CS-PEG-Folate constructs did not significantly decrease MDR1 translation in comparison to the NH_2_-MSN-siRNA. This was due to the low expression of folate receptors on the surface of these cells, which led to nonspecific and lower uptake of nanoparticles. According to the literature, both TAT and chitosan have proton sponge properties and several studies reported that chitosan has a lower proton sponge capacity and transfection^43–46^. TAT and chitosan enter cells via a nonspecific uptake mechanism that is dependent on the nature of the cell membrane and varies from cell to cell. On the contrary, targeted dual nanostructures have a higher concentration of PEG molecules on their surface, which may hinder cell entry and endosomal escape in NH_2_-MSN-siRNA-CS-PEG-folate/TAT structure.

## Conclusion

In this study, a dual tumor-targeting design of MSNs for gene delivery was successfully fabricated. The aminated MSNs with medium pore sizes of approximately 5 nm and a surface area of 512 m^2^/g demonstrated a high siRNA loading capacity. Folate and TAT were attached to chitosan using a small PEG spacer to target the cancer cells and improve the solubility. The functionalized chitosan effectively covered the surface of NH_2_-MSN-siRNA and protected the siRNA cargo within NH_2_-MSNs from enzymatic degradation. Flow cytometry showed the folate conjugated nanocarriers could bind to folate receptor-rich HeLa-RDB as the target cells with higher efficiency than simple MSNs and chitosan-MSNs. TAT also increased the cellular internalization of nanocarriers in both HeLa-RDB and EPG85.257-RDB cells. siMDR1 delivery decreased MDR1 transcription and translation in HeLa-RDB and EPG85.257-RDB cells, but the dual tumor-targeting design of NH_2_-MSNs demonstrated the greater silencing ability of P-gp in HeLa-RDB cells. The smart nanoparticles demonstrated enhanced anti-cancer activity of daunorubicin drugs due to P-gp knockdown in HeLa-RDB cells. Our findings suggest that NH_2_-MSN-siRNA-chitosan-PEG-folate/TAT may be a promising nanocarrier for targeted siMDR1 delivery into cancer cells resistant to P-gp inhibition.

## Materials and methods

### Materials

Folic acid, thioglycolic acid, 1-Ethyl-3-(3-dimethylaminopropyl) carbodiimide (EDC), *N*-hydroxy succinimide (NHS), polyethylene glycol (PEG), Cetyltrimethylammonium chloride (CTAB), tetraethyl orthosilicate (TEOS), 1,3,5-trimethylbenzene (TMB), and (3-aminopropyl) triethoxysilane (APTES) were purchased from Sigma-Aldrich. The Human ABCB1 siRNA and the negative control siRNA were purchased from Bioneer Corporation (South Korea). Optimized TAT peptide was ordered from TAG-Copenhagen (Copenhagen, Denmark). All other reagents and solvents were of analytical grade and provided by Sigma-Aldrich (Deisenhofen, Germany). HeLa cells (MDR1 negative and high folate receptor-expressing line) and EPG85.257-RDB (high MDR1 and trace folate receptor-expressing line) were kindly supplied by Professor Herman Lage (Universitäts medizin Berlin, Germany).

### MSN synthesis and surface amination

NH_2_-MSNs with large pores were prepared in the presence of TMB as a micelle swelling agent. Initially, 1 g CTAB was dissolved in 480 ml deionized water, the temperature of the mixture was increased to 60 °C, 7 ml TMB was added, and the mixture was stirred for 5 h. The solution was then poured with 3.5 ml of 2 M sodium hydroxide. Following that, the temperature was increased to 80 °C, and 5 ml TEOS and 0.2 ml APTES were simultaneously inset dropwise into the solution for 5 min, followed by another 2 h of stirring. After cooling, the white precipitate was washed with generous amounts of 96% ethanol, deionized water, and freeze-dried. By incorporating APTES into the silanization process, the pores and surface were functionalized with amine groups. Additionally, the outer surface of the nanoparticles was covalently aminated. Afterward, 1 g of nanoparticles was dispersed in 100 ml ethanol containing 2% v/v APTES and stirred for 12 h at 60 °C under reflux conditions. NH_2_-MSNs were collected with centrifugation at 14000 rpm for 15 min at ambient temperature. Finally, 1 g nanoparticle was resuspended in 200 ml 96% ethanol containing 2 g NH_4_NO_3_, and the suspension was refluxed at 60 °C for 12 h. The precipitate was collected via centrifugation, and the process was repeated at least three times to remove the surfactant from the pores completely. For future use, the surfactant-free NH2-MSNs were freeze-dried and stored in 96% ethanol^47^. A transmission electron microscope (TEM, Zeiss, Germany), a scanning electron microscope (SEM, JEOL, Japan), X-ray diffraction (XRD, STOE, Germany), and Fourier transform infrared spectroscopy were used to physiochemically examine the nanoparticles (FTIR, Bruker, Germany). A nitrogen adsorption analyzer was used to determine the surface area (Belsorp-mini, Japan). All samples' particle size and zeta potential were determined in deionized water using a DLS (Malvern, Worcestershire, England).

### siRNA loading and gel retardation assay

Through RNA retardation assay, the siRNA loading capacity was optimized. For 30 min at room temperature, siRNA and aminated-MSN were mixed at various ratios (naked siRNA, 1:0.5, 1:1, 1:2, 1:3, 1:4, 1:5, 1:6, 1:7 μg/μg in 50 μl water). The RNA-MSN complex was loaded with a dye, and electrophoresis was performed on a 1.5% agarose gel containing Gel-Red at a constant 80 V for 30 min. The migration patterns were recorded at 254 nm irradiation^27^. The loading capacity was determined using the following formula:

$${\text{Loading}}\;{\text{capacity}}\;\% = \frac{{Weight\;of\;loaded\;siRNA}}{{Weight\;of\;the\;nanoparticles}} \times 100$$

### Covalent pegylation of TAT and folate

First, folate was thiolated and activated for nucleophilic substitution using 2-mercaptoacetic acid. Then, 2 mg SH-folate and 7 mg MAL-PEG(3000)-NHS was stirred for 24 h at room temperature in the dark under argon protection in 1 ml anhydrous DMSO (1:1 molar ratio).This reaction results in the formation of folate-MAL-PEG(3000)-NHS. Second, a cysteine modified version of TAT peptide (HS-CYGRKKRRQRRR-NH_2_) was linked to MAL-PEG(3000)-NHS to form TAT-MAL-PEG(3000)-NHS. Briefly, 1 mg TAT and 2 mg MAL-PEG(3000)-NHS (5:1 molar ratio) was stirred in 1 ml deionized water for 24 h at 4 °C. The reaction was controlled on TLC (silica gel-60 F254) using methanol, chloroform, and water (45:9:1). TLC was stained with Dragendorff'sreagent and ninhydrin. For both reactions, unreacted substrates were dialyzed (3.5 kD, Sigma-Aldrich) over deionized water^32^. Conjugation efficiency for the reactions was calculated through the Ellman assay for free sulfhydryl measurement: 100 × ((substrate-SH) − (PEG-S-substrate)) ÷ substrate-SH). Finally, pure conjugates were lyophilized and maintained at a temperature of 4 °C^48^.

### Chitosan modification and targeting

Four sequential syntheses were used to generate MSN targeting coats. Amine moieties on chitosan were determined using ^1^H-NMR in a solution containing 2% deuterated acetic acid.The pH was adjusted to 6.0 after dissolving 10 mg chitosan (equivalent to 40 mol NH2 groups) in 2 ml of 2% acetic acid. Polyethylene glycol, folate, TAT, or double folate/TAT were used to conjugate chitosan. Around 0.8 mol of MAL-PEG(3000)-NHS, TAT-PEG(3000)-NHS, folate-PEG(3000)-NHS, or a mixture of TAT-PEG(3000)-NHS and folate-PEG(3000)-NHS was added to 2 ml chitosan solution (1:50 ratio to amine group) and stirred in the dark at room temperature. After 3 h, the pH was raised to 7 for another 24 h. For 48 h, the solution was dialyzed against deionized water (cutoff 12 kD, SLS, UK). The modified chitosans included chitosan-PEG(3000), chitosan-PEG(3000)-folate, chitosan-PEG(3000)-TAT, chitosan-PEG(3000)-folate/TAT and were collected after freeze-drying and stored at 4 °C. The conjugations were confirmed and quantified using NMR^49–51^.

### Formulation and evaluation of MSNs coated with functionalized chitosan

NH_2_-MSNs loaded with optimized RNA were briefly coated with various functionalized chitosans, and 5 µl of the MSN stock (0.1% w/v) plus 25ρmol of siRNA were stirred gently for 30 min to form the NH_2_-MSN-siRNA complex. Then, 2 µl of each functionalized-chitosan solution (0.5% w/v in 2% acetic acid, pH: 6) was added to the RNA-loaded NH_2_-MSNs and stirred for 24 h. The chitosan-coated NH_2_-MSN-siRNA were collected by centrifugation (14000 rpm, 15 min) and washed twice with deionized water^52^. Zeta potential was measured to verify the chitosan coating on the surface of the MSN structures. Finally, the protective role of chitosan coats was evaluated by incubating the nanostructures with 0.25% RNase-A at optimal conditions for varying times. The enzyme was inactivated at 60 °C for 5 min. Heparin (200 IU/µl) was added to the complexes for 10 min to release the siRNA, and agarose gel electrophoresis was performed to identify the siRNA degradation level^27,42,53^. For the stability of nanoparticles, siRNA leakage from nanoparticles was studied for 4 days in PBS by UV-spectroscopy at 260 nm. Briefly, the siRNA loaded nanoparticles were dispersed in PBS at 4 °C. At a predetermined sampling time (1, 2, 3, and 4 day), the suspension was centrifuged and samples were taken from the supernatant. siRNA quantities of the supernatant were determined by UV-spectroscopy at 260 nm.

### HeLa transfection with multidrug-resistant pumps

The cell lines were cultivated in RPMI-1640 medium supplemented with 10% fetal bovine serum (FBS), 100 mg/ml streptomycin, and 100 IU/ml penicillin at 37 °C in a 5% CO2 atmosphere. Multidrug-resistant HeLa-RDB was generated by transducing HeLa cells using engineered lentiviral particles with the human ABCB1 (NM 000927.4) gene (pReceiver-Lv152 plasmid, CMV promoter, hygromycin resistant, Genecopoeia Company). HeLa cells (5 × 10^4^ cells/well) were cultured in 24-well plates. After achieving 75% confluence, recombinant lentiviruses were added at multiplicity of infection (MOI) values ranging from 0.5 to 10. After 24 h, the virus-containing medium was removed from the wells and replaced with a fresh medium. The cells were treated with an optimum hygromycin-B concentration that kills untransfected cells to select stably transduced cells. The drug-resistant colonies of HeLa cells became visible after 10 days. EPG85.257, HeLa, and their MDR1 resistant counterparts were evaluated for folate receptor and MDR1 pumps using real-time PCR^48^.

### Growth pattern and functional cytotoxicity kinetics

Recombinant and parental cell lines were seeded at a density of 1000 cells per well in 96-well plates to measure the growth kinetics during 7 days. Cellular proliferation was monitored daily using MTT. A trend line was depicted on natural logarithms of the cell number (dependent variant) versus times (independent variant). According to the Monod equation in the logarithmic phase, the cell-specific growth rate (μmax) was considered the slope of the line. The cell cytotoxicity of the final nanostructures was determined on HeLa-RDB cells and EPG85.257-RDB cells. In 96-well plates, 3000 cells were seeded per well. Cells were treated with various concentrations of nanoparticles (0, 20, 60, 100, 140, and 180 µg/ml) after 24 h. After 72 h of treatment, optical density at 570 nm was used to determine the viable cell fraction in the MTT assay. Finally, daunorubicin toxicity (0–10000 nm) was determined in the presence of siRNA-loaded nanoparticles (10 µg/ml) to assess whether the multidrug-resistant cells were chemosensitized. The IC_50_ value was defined as the concentration of daunorubicin required to inhibit cell growth by 50% in the presence of silencing RNA^39,48^.

### Nanoparticle endocytosis and competition analysis

Cellular uptake was assessed by flow cytometry (CyFlow Space^®^, Munster, Germany) using the FL-2 channel. HeLa-RDB and EPG85.257-RDB cells (2 × 10^5^ cells per well) were seeded into 12-well plates. Initially, 10 μl of DiI fluorescent dye (1 mg/ml in ethanol) was loaded into 10 mg of NH_2_-MSN. The DiI-MSNs were washed three times with ethanol and centrifuged to collect them. The fluorescent nanosilica particles were then coated with various modified chitosans, and cells were treated for 2 h at 37 °C with 100 μg/ml of the modified-fluorescence nanovehicles^39^. After washing the cells with PBS, the mean intracellular fluorescence intensity indicated the constructs' endocytosis activity. The same procedure was used to conduct a competitive uptake assay in the presence and absence of 5 µM folate as the competitor. Data were analyzed using FlowJo software (version 7.6.1) and compared to the control samples (treated cells with uncoated DiI-NH_2_-MSN)^54^.

### Molecular evaluation of gene silencing

Real-time PCR and Western blotting were used to determine the MDR1 level. In 6-well plates, HeLa-RDB and EPG85.257-RDB cells were seeded. After 24 h, cells were transfected with optimized si-MDR1-loaded nanovehicles (25 pmol si-MDR1/5 µg MSNs) coated with specific chitosans. After 48 h, RNA was extracted, cDNA was synthesized, and real-time PCR was performed according to the Pfaffl method on a Rotor-GeneQ instrument (Qiagen, Germany) using the SYBR Green kit (Qiagen kits, Germany). Simultaneously, proteins were extracted using a lysis buffer (8 M urea, 2 M thiourea, Tris 10 mM, pH = 8.0), and the Bradford method was used to quantify the proteins colorimetrically. Proteins were run in a 12% SDS-PAGE according to the Laemmli method and then electroblotted to the nitrocellulose membrane using a semi-dry Trans-Blot apparatus (Bio-Rad, Richmond, CA). Blots were briefly blocked by 5% skimmed milk, incubated with a primary anti-Pgp mouse monoclonal IgG (1:500 v/v) overnight at 4 °C, treated with an HRP-conjugated secondary antibody (1:5000 v/v) for 2 h, and photos were taken using the luminal system on a blot scanner (LiCor, Lincoln, NE). For quantification procedures, β-actin was considered as the internal normalizer for both quantifications^48^.

### Statistical analyses

Results were expressed as mean ± standard deviation of at least three independent experiments and analyzed using graph pad prism. P values < 0.05 were considered statistically significant.

### Ethical statement

The authors have read and adhered to the journal's ethical standards for manuscript submissions. This article does not contain any studies with human participants or animals performed by any of the authors. We declare that the submitted manuscript does not contain previously published materials and is not considered for publication elsewhere. All the authors have contributed to conception and design, collection, analysis, and interpretation of data, writing or revising the manuscript, or providing guidance on the research's execution.

## Supplementary Information

Supplementary Information.

## Data Availability

The datasets generated or analyzed during the current study are available on request from the corresponding author.
