# Supplementary material for: siRNA delivery using intelligent chitosan-capped mesoporous silica nanoparticles for overcoming multidrug resistance in malignant carcinoma cells
Source: Sci Rep. 2021 Oct 15;11:20531. doi: 10.1038/s41598-021-00085-0 (PMC8519957; doi:10.1038/s41598-021-00085-0)
Supplement: Supplementary file 1 — Supplementary Information. [file 41598_2021_85_MOESM1_ESM.pdf]

Heidari et al **Supplementary Dataset**

Stimuli-responsive siRNA delivery using intelligent chitosan-capped mesoporous silica nanoparticles for overcoming multidrug resistance in malignant carcinoma cells

Running title:

Targeted gene delivery to overcome drug resistance

Razieh Heidari [1], Pegah Khosravian (PharmD, PhD) [2], Seyed Abbas Mirzaei (PharmD, PhD) [1,3], Fatemeh Elahian (PharmD, PhD) [1,3*]

1. Department of Medical Biotechnology, School of Advanced Technologies, Shahrekord University of Medical Sciences, Shahrekord, Iran.

2. Medical Plants Research Center, Basic Health Sciences Institute, Shahrekord University of Medical Sciences, Shahrekord, Iran

3. Cellular and Molecular Research Center, Basic Health Sciences Institute, Shahrekord University of Medical Sciences, Shahrekord, Iran.

Author for Correspondence:

F. Elahian, PharmD, PhD

Cellular and Molecular Research Center, Basic Health Sciences Institute,

Shahrekord University of Medical Sciences, Shahrekord, Iran

Tel: (+98) 38 33331471; Fax: (+98) 38 33330709

E-mail: elahian@skums.ac.ir, dr.elahian@yahoo.com

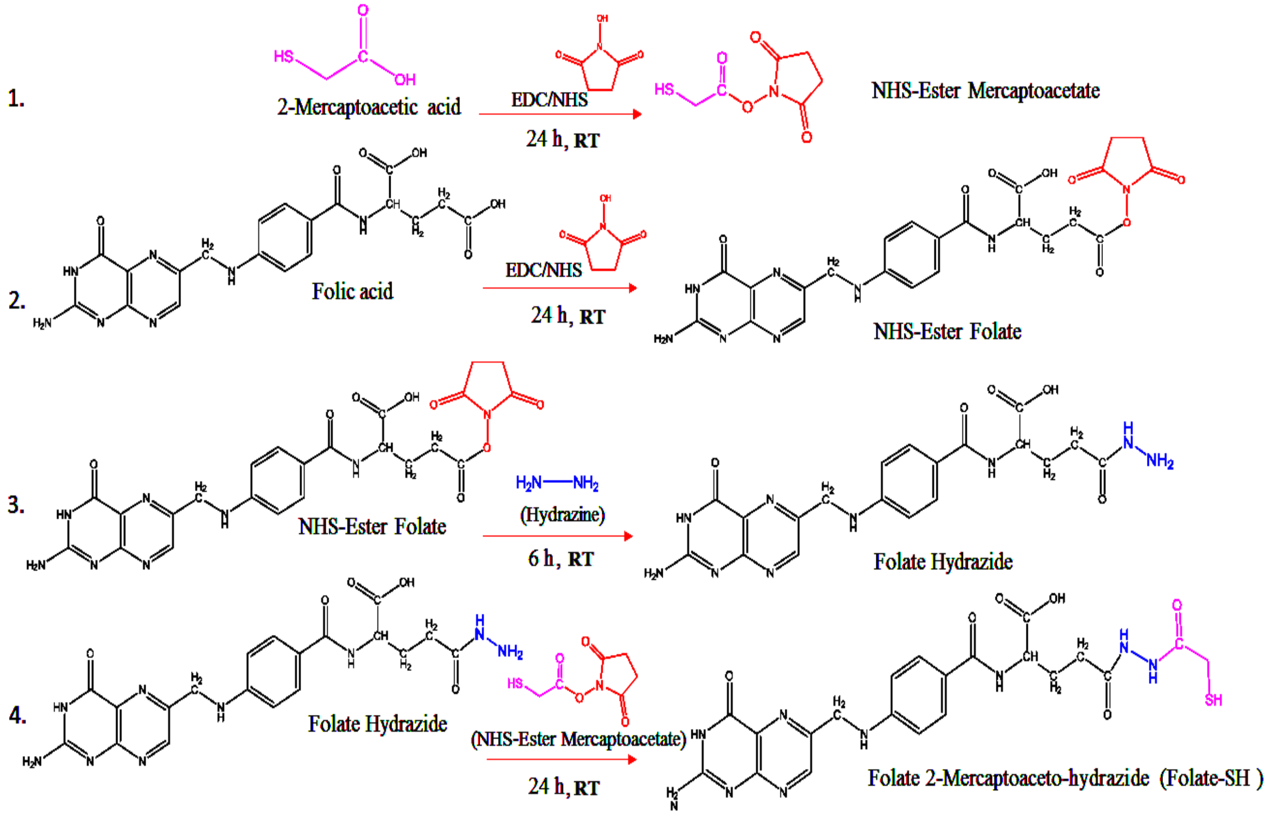

Figure S1. Steps involved in synthesis of folate-SH.

1) NHS-ester synthesis of the 2-Mercaptoacetic acid. The carboxylic acid group of the 2-Mercaptoacetic acid (22 µl) was activated via an EDC/NHS coupling reaction in 0.5 ml DMSO together (molar ratio 2:2).

2) 200 mg Folic acid was dissolved in 4 ml anhydrous DMSO together with 57.5 mg NHS and 95 mg EDC (molar ratio 1:1) and then continuously stirred for 24 h in the dark under argon atmosphere.

3) 114 µl Hydrazine hydrate was added into the NHS-ester folate (molar ratio 1:5) under stirring for 6 h under similar conditions. Folate-hydrazide salt was formed by adding 1.2 ml HCl (0.5 N) to the reaction. The product was precipitated by adding 22 ml acetone/ether (30% v/v) for overnight then the pellet was centrifuged. The yellow precipitate was re-suspended with a small volume of distilled water and re-precipitated whit 20 ml 96% ethanol for overnight in -20 °C. Yellow particles were centrifuged and washed three times with 96% ethanol and ether. Precipitates were lyophilized and stored in the dark at -20 °C. The yield was 64.7%.

4) Finally, 150 mg folate-hydrazide solution was dissolved in 1 ml DMSO, and then the NHS ester of 2-mercaptoacetic acid solution was added to the folate-hydrazide solution (molar ratio 1.5:1) and stirred for 12 h at room temperature. The reaction yield was 70.7 ± 5.5%. NHS: N-hydroxysuccinimide, EDC: Carbodiimide, RT: Room Temperature.

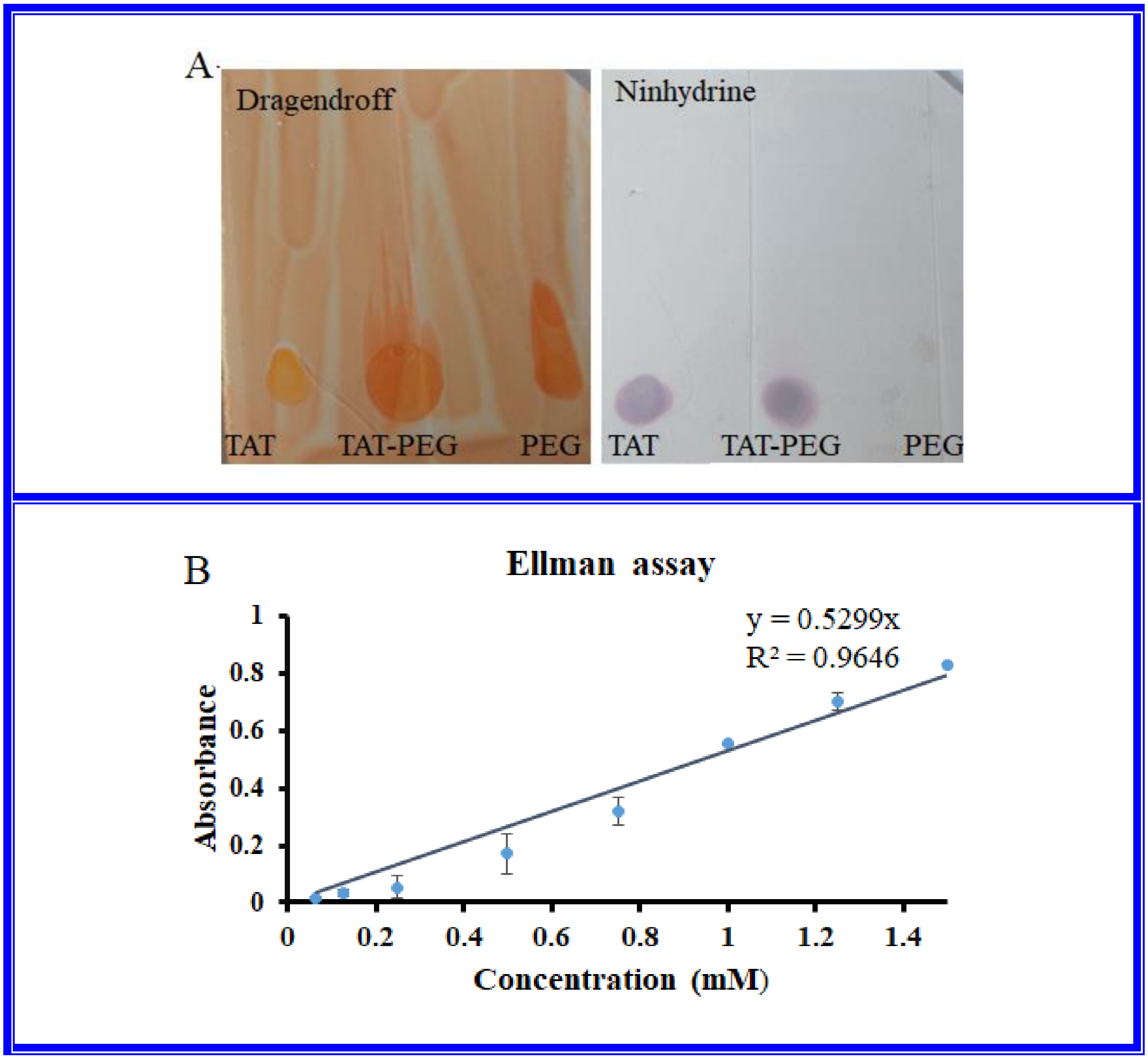

Figure S2. (A) Thin-layer chromatography of TAT-PEG (3000) structures with ninhydrin and dragendorff's reagents to visualize of the TAT and PEG polymer respectively. (B) Ellman's assay was used for quantification of free sulfhydryl groups in solution before the separation of unreacted SH-folate and SH-TAT according to the manufacturer's instruction. In brief, different concentrations of cysteine hydrochloride monohydrate (0.05 to 1.5 mM) were dissolved and plotted as a standard curve with y=0.5299x equation and squared regression coefficient of 0.96. Ellman's reagent was prepared by dissolving 4 mg of Ellman's reagent in 1 ml reaction buffer. Reaction buffer composed of sodium phosphate (100 mM) and EDTA (1 mM,) at pH= 8.0. To quantify the free substrate-SH in reaction, 50 µl Ellman's reagent solution and 2.5 ml reaction buffer was added to 250 µl of the sample. Then reaction was incubated 15 min at 37 °C and the

absorbance was recorded at 412 nm using UV-visible spectrophotometer. Degree of substitution SH-folate and SH-TAT on PEG (3000) was calculated according to the following equation: Conjugation efficiency = 100 × ((substrate-SH) – (PEG-S-substrate)) ÷ substrate-SH). Conjugation efficiency of the SH-folate and Cys-TAT to the maleimide group of MAL-PEG(3000)-NHS were determined to be 86.5% and 70.2%, respectively.

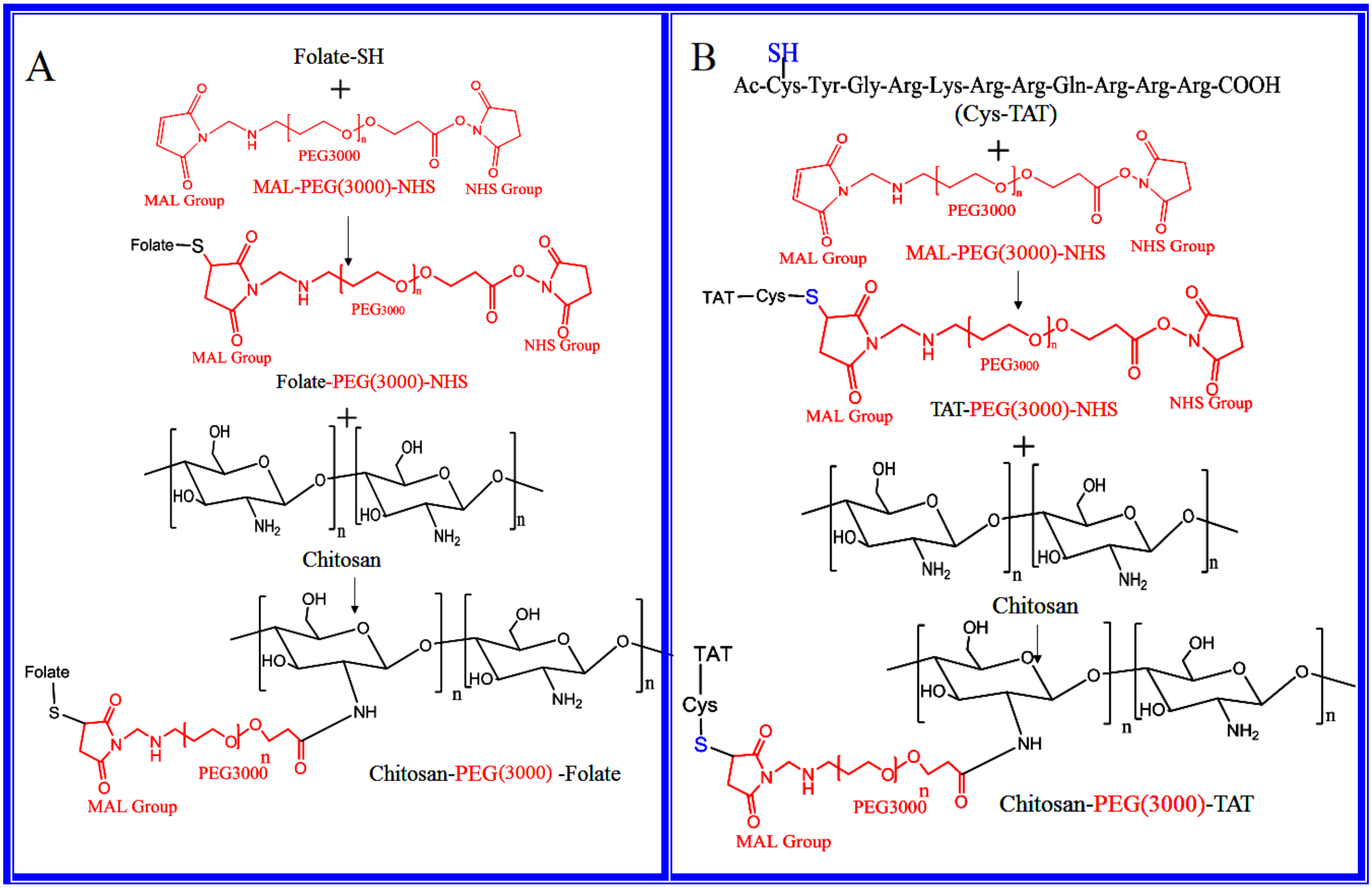

Figure S3. Schematic synthesis of chitosan-PEG(3000)-folate (A) and chitosan-PEG(3000)-TAT (B). NHS: N-Hydroxysuccinimide, PEG: polyethylene glycol, TAT: trans-activator of transcription.

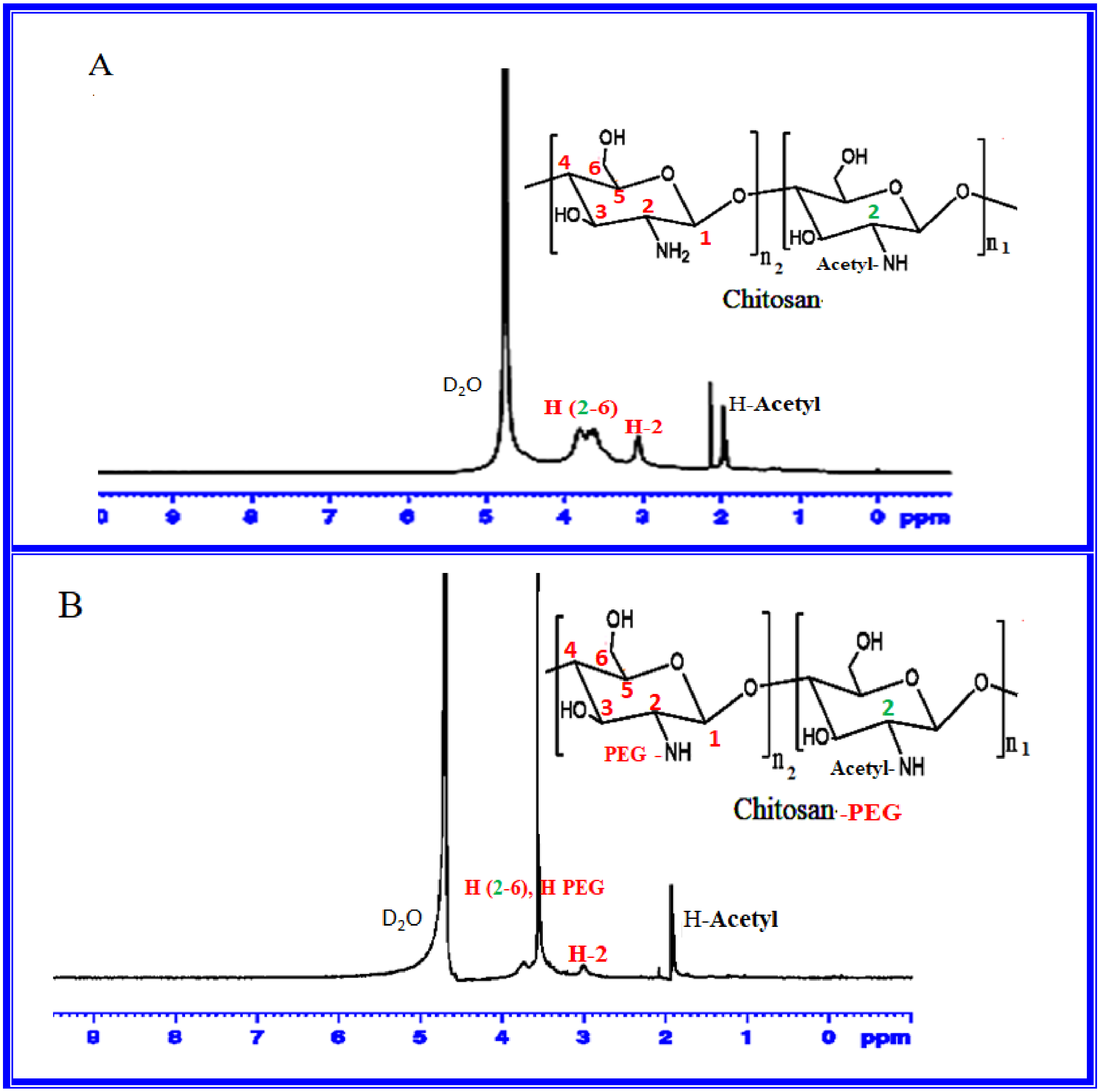

Figure S4. ^{1}H-NMR spectrum of chitosan and chitosan-PEG.

(A) ^{1}H-NMR spectrum of chitosan showed a typical peaks of chitosan including: 1.9-2 ppm peaks corresponding to the resonance of the protons of N-acetyl glucosamine (GlcNAc) residues and the peak at 3.3-4 ppm corresponding to the H_2-H_6 and the peak at 3.1-3.2 ppm corresponding to the H_2 proton of glucosamine (GlcN). The degree of acetylation (DA) determined 25% by

integration of the singlet peak at 1.9-2 ppm against the multiples peak between 3.3-4 ppm according to:

$$DA= \left(\frac{\frac{1}{3} I\,CH3}{\frac{1}{6} I\,H2-H6}\right)\times 100 \text{ equation.}$$

(B) ^{1}H-NMR spectra of chitosan-PEG, the enhanced peaks at 3.3-4 ppm associated with the PEG methylene groups. The degree of substitution (DS) of PEG on the chitosan were obtained 1.7% by integration of the 2.06 ppm (corresponding to the acetyl group) and the multiples peaks at 3.1-4.3 ppm (corresponding to the PEG and H_2-H_6 signals of chitosan according to the following equation. H_{PEG}: Number of protons in PEG.

$$DS_{PEG}= \frac{(IH2-H6,HPEG)-(\frac{6ICH3}{3DA}\times 100)}{\frac{I\,CH3}{3\,DA}\,HPEG\times 100} \times 100$$

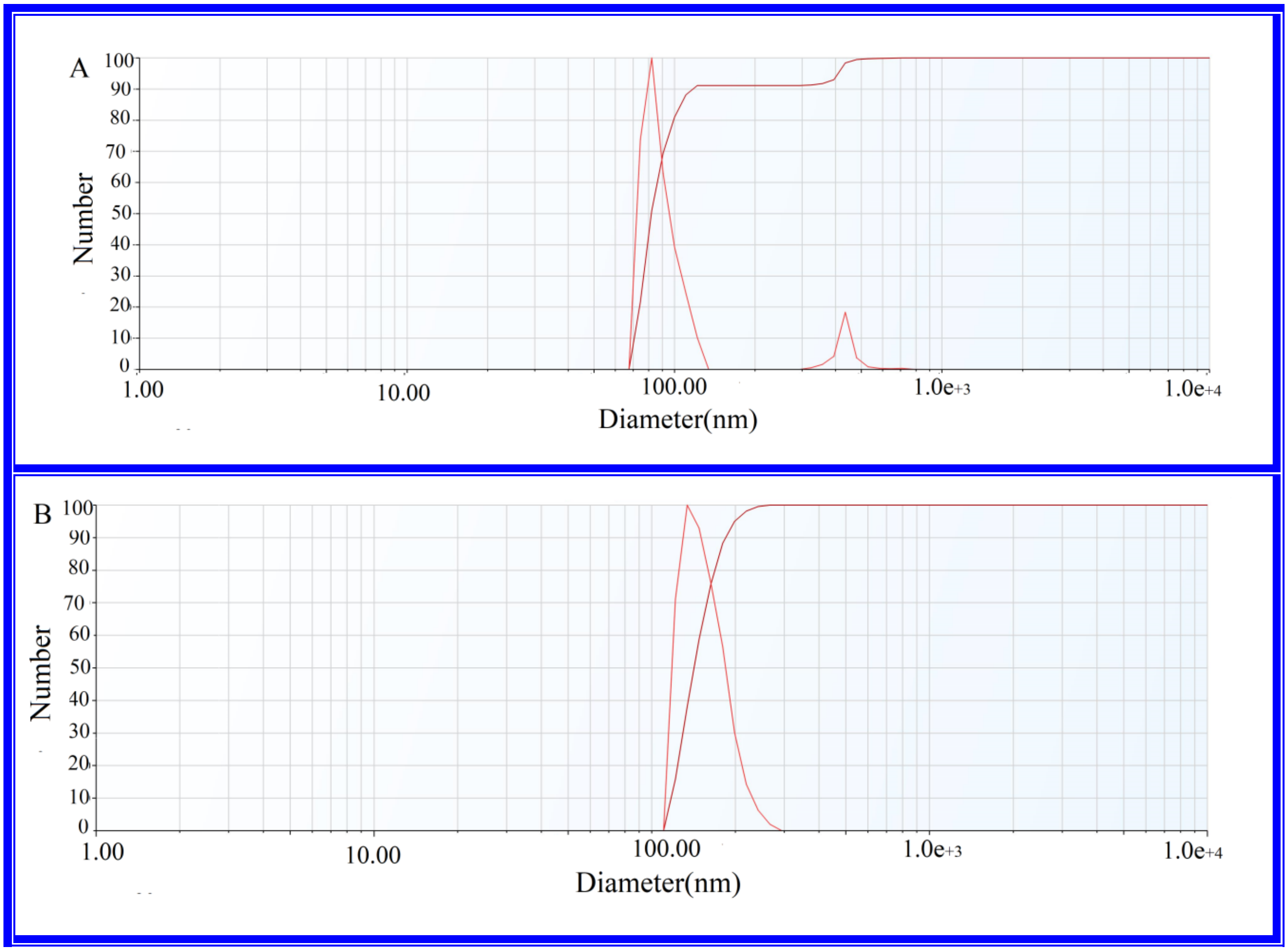

Figure S5. Dynamic light scattering (DLS) results of NH_2-MSN (A) and NH_2-MSN-siRNA coated with chitosan-PEG-folate/TAT (B). Nanoparticles around 100 nm are the DLS results.

A

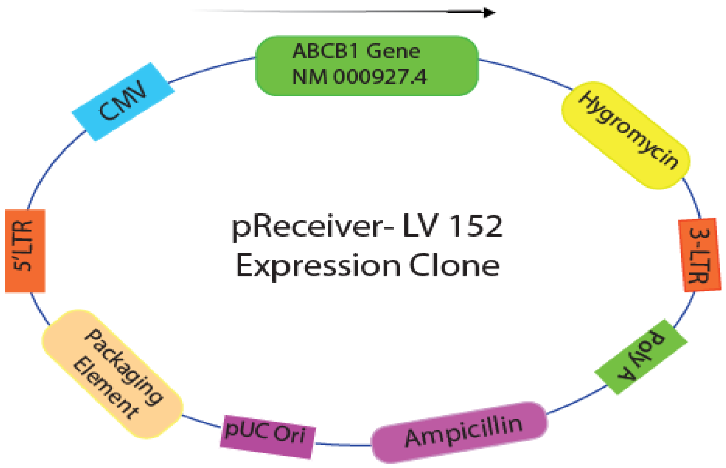

B

```
MDLEGDRNGGAKKKNFFKLNNKSEKDKKEKKPTVSVFSMFRYSNWLDKLYMVVGTLAAIIHGAGLPLMML
VFGEMTDIFANAGNLEDLMSNITNRSDINDTGFFMNLEEDMTRYAYYYSGIGAGVLVAAYIQVSFWCLAA
GRQIHKIRKQFFHAIMRQEIGWFDVHDVGELNTRLTDDVSKINEGIGDKIGMFFQSMATFFTGFIVGFTR
GWKLTLVILAISPVLGLSAAVWAKILSSFTDKELLAYAKAGAVAEEVLAAIRTVIAFGGQKKELERYNKN
LEEAKRIGIKKAITANISIGAAFLLIYASYALAFWYGTTLVLSGEYSIGQVLTVFFSVLIGAFSVGQASP
SIEAFANARGAAYEIFKIIDNKPSIDSYSKSGHKPDNIKGNLEFRNVHFSYPSRKEVKILKGLNLKVQSG
QTVALVGNSGCGKSTTVQLMQRLYDPTEGMVSVDGQDIRTINVRFLREIIGVVSQEPVLFATTIAENIRY
GRENVTMDEIEKAVKEANAYDFIMKLPHKFDTLVGERGAQLSGGQKQRIAIARALVRNPKILLLDEATSA
LDTESEAVVQVALDKARKGRTTIVIAHRLSTVRNADVIAGFDDGVIVEKGNHDELMKEKGIYFKLVTMQT
AGNEVELENAADESKSEIDALEMSSNDSRSSLIRKRSTRRSVRGSQAQDRKLSTKEALDESIPPVSFWRI
MKLNLTEWPYFVVGVFCAIINGGLQPAFAIIFSKIIGVFTRIDDPETKRQNSNLFSLLFLALGIISFITF
FLQGFTFGKAGEILTKRLRYMVFRSMLRQDVSWFDDPKNTTGALTTRLANDAAQVKGAIGSRLAVITQNI
ANLGTGIIISFIYGWQLTLLLLAIVPIIAIAGVVEMKMLSGQALKDKKELEGSGKIATEAIENFRTVVSL
TQEQKFEHMYAQSLQVPYRNSLRKAHIFGITFSFTQAMMYFSYAGCFRFGAYLVAHKLMSFEDVLLVFSA
VVFGAMAVGQVSSFAPDYAKAKISAAHIIMIIEKTPLIDSYSTEGLMPNTLEGNVTFGEVVFNYPTRPDI
PVLQGLSLEVKKGQTLALVGSSGCGKSTVVQLLERFYDPLAGKVLLDGKEIKRLNVQWLRAHLGIVSQEP
ILFDCSIAENIAYGDNSRVVSQEEIVRAAKEANIHAFIESLPNKYSTKVGDKGTQLSGGQKQRIAIARAL
VRQPHILLLDEATSALDTESEKVVQEALDKAREGRTCIVIAHRLSTIQNADLIVVFQNGRVKEHGTHQQL
LAQKGIYFSMVSVQAGTKRQ
```

Figure S6. Lentiviral particles were recombinated with MDR1-pReceiver LV152 plasmids (A). Human ABCB1 gene was extracted from NCBI (NM_000927.4). It is cloned under CMV promoter in the pReceiver-Lv152 plasmid contains a hygromycin-B resistant. (B) MDR1 Protein sequence (NP_000918.2) contains 1280 amino acids.

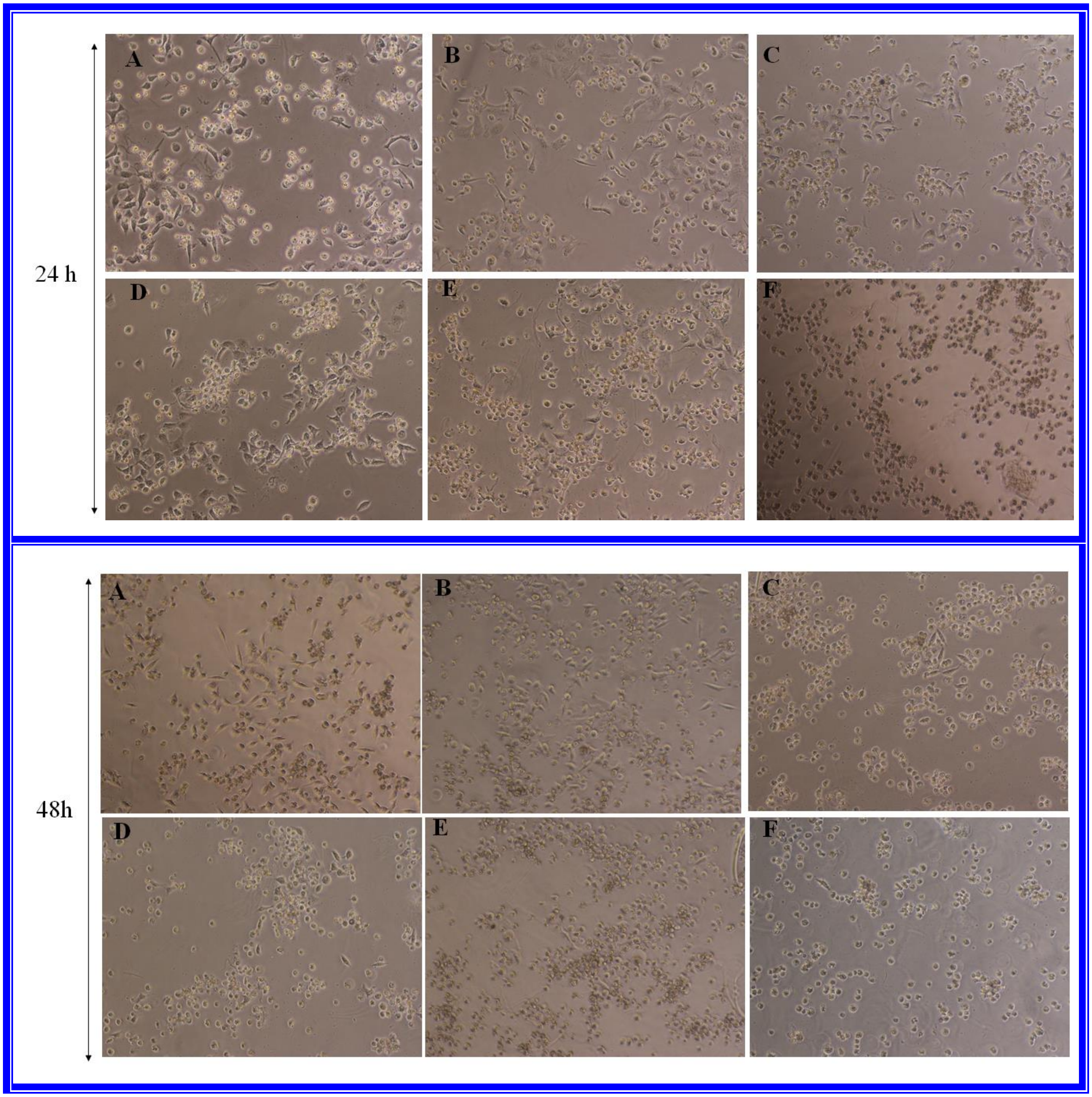
24 h
A
B
C
D
E
F
48h
A
B
C
D
E
F

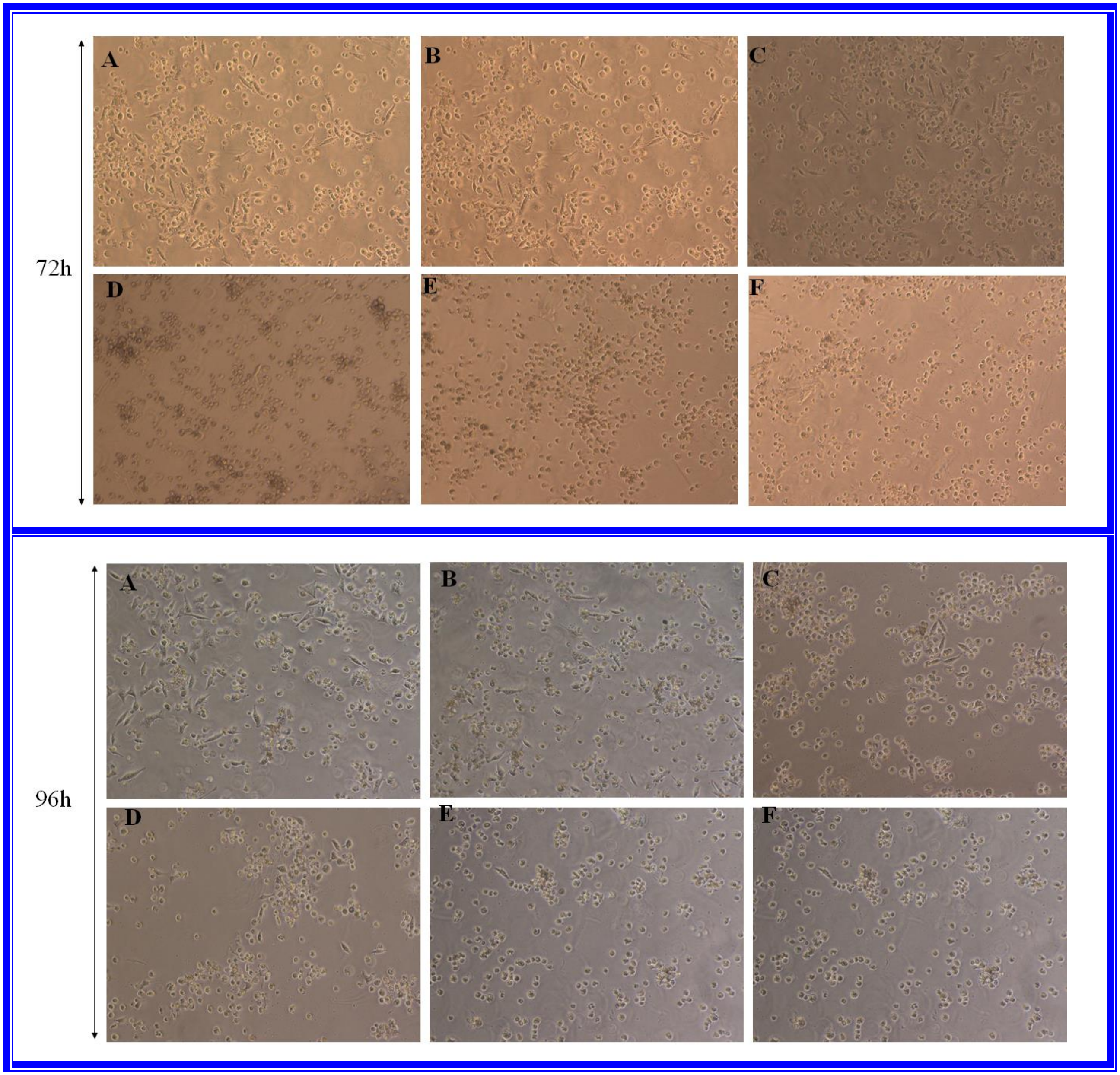
72h
A
B
C
D
E
F
96h
A
B
C
D
E
F

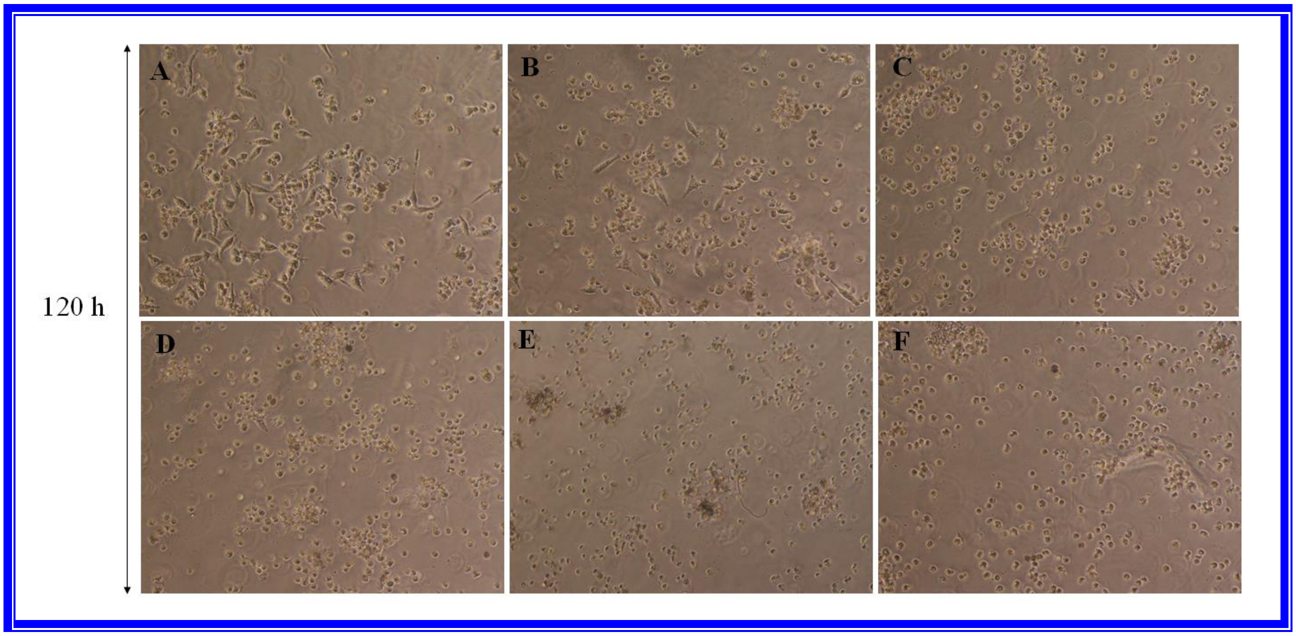

Figure S7. Microscopic images of HeLa cells after treatment with different concentrations of hygromycin (A: 50, B: 100, C: 200, D: 300, E: 400 and F: 500 µg/ml) for 5 days. The minimum lethal dose of hygromycin was determined 200 µg/ml after 120 h to select transfected colonies. Cells were pictured with 100X magnification.

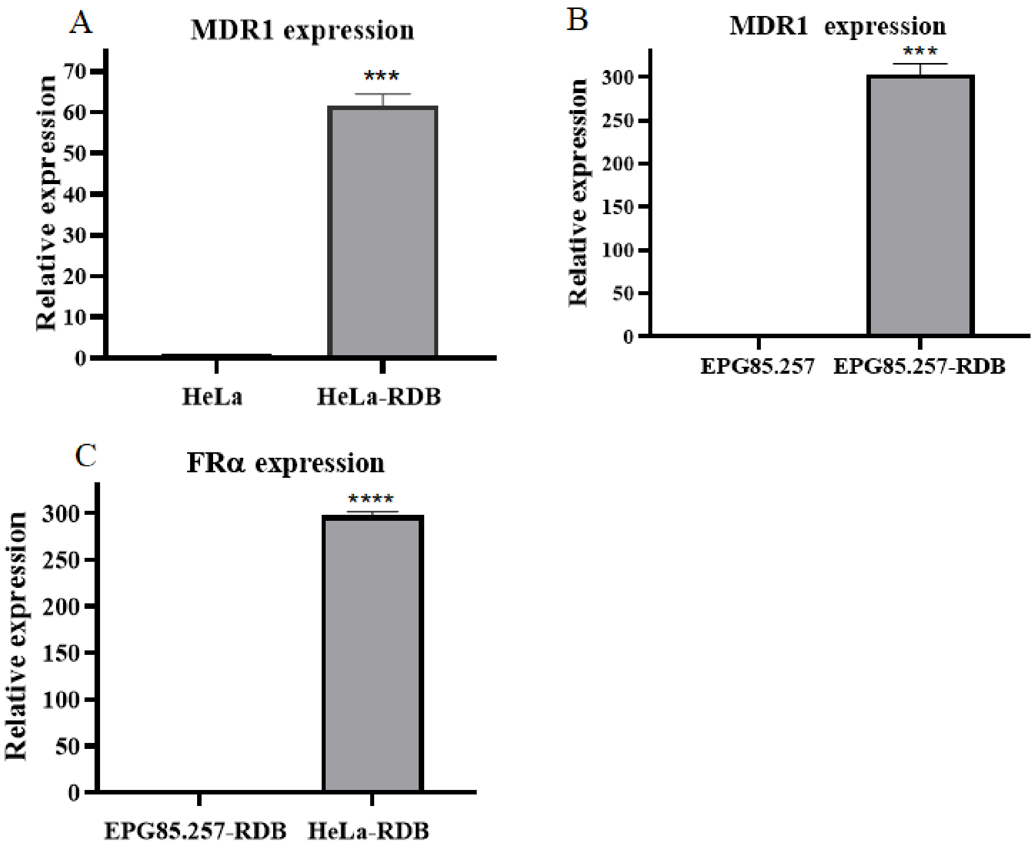

Figure S8. Real-time PCR results of MDR1 and folate receptor-α (FRα) expression. (A) HeLa-RDB cells that engineered with MDR1 expressed the gene 58.4 folds more than HeLa cells. (B) EPG85.257-RDB expresses MDR1 gene 310.4 folds more than EPG85.257. (C) Folate receptor-α expression in HeLa-RDB was recorded 297.1 folds more than EPG85.257-RDB. Symbols (*** and ****) describe significant differences between the control and treatment samples by $p<0.001$ and $p<0.0001$, respectively.

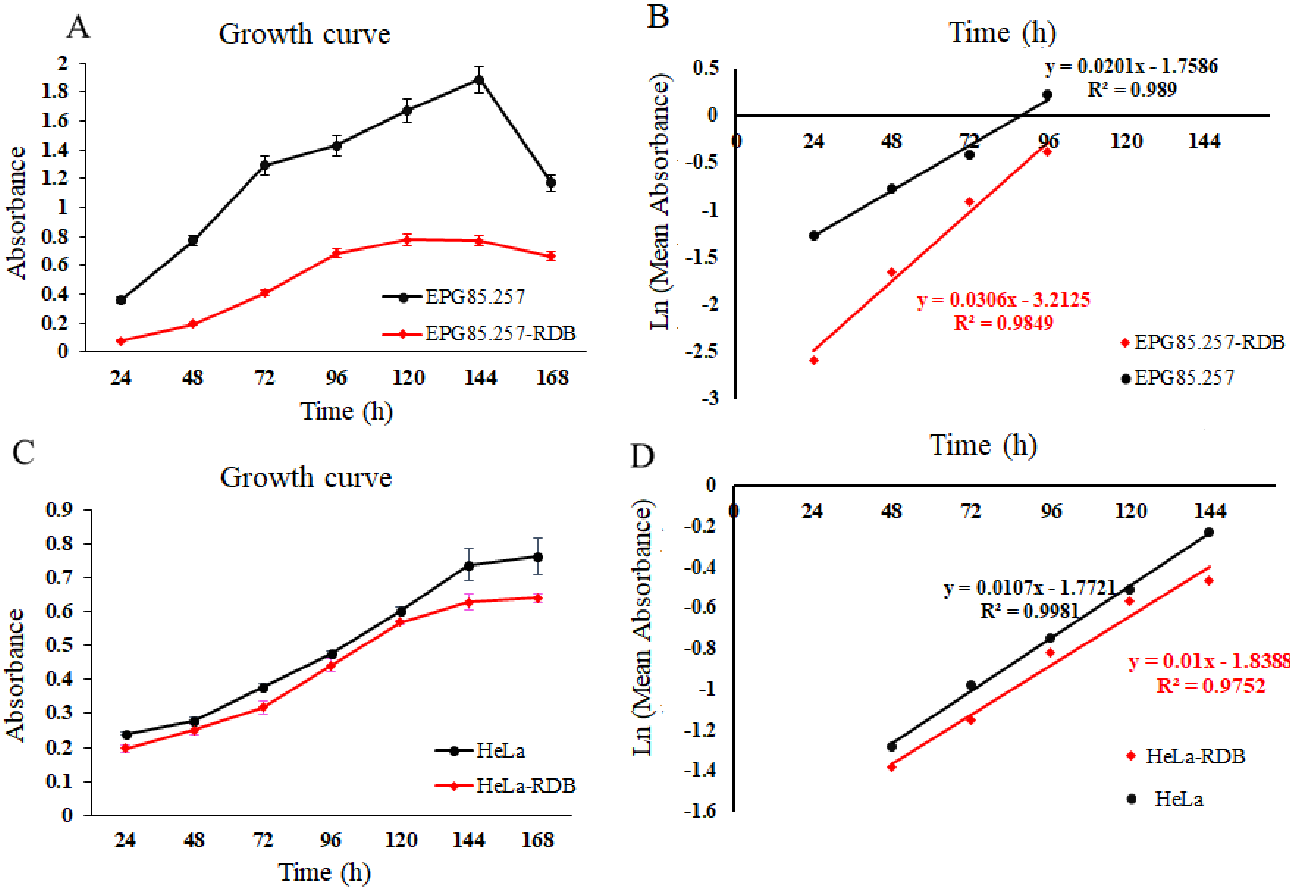

Figure S9. Growth kinetics and doubling time analyses for EPG85.257, EPG85.257-RDB (A, B), HeLa and HeLa-RDB cells (C, D). All curves were fitted to linear trend line and line slopes represent maximum specific growth rates (μ) according to the Monod equation in the logarithmic phase. Maximum specific growth rates (μ) were calculated 0.0201, 0.0306, 0.0107 and 0.0100 for EPG85.257, EPG85.257-RDB, HeLa and HeLa-RDB cells respectively. Doubling time of EPG85.257, EPG85.257-RDB, HeLa, and HeLa-RDB cells were obtained 34.5, 22.6, 64.8 and 69.3 h, respectively.

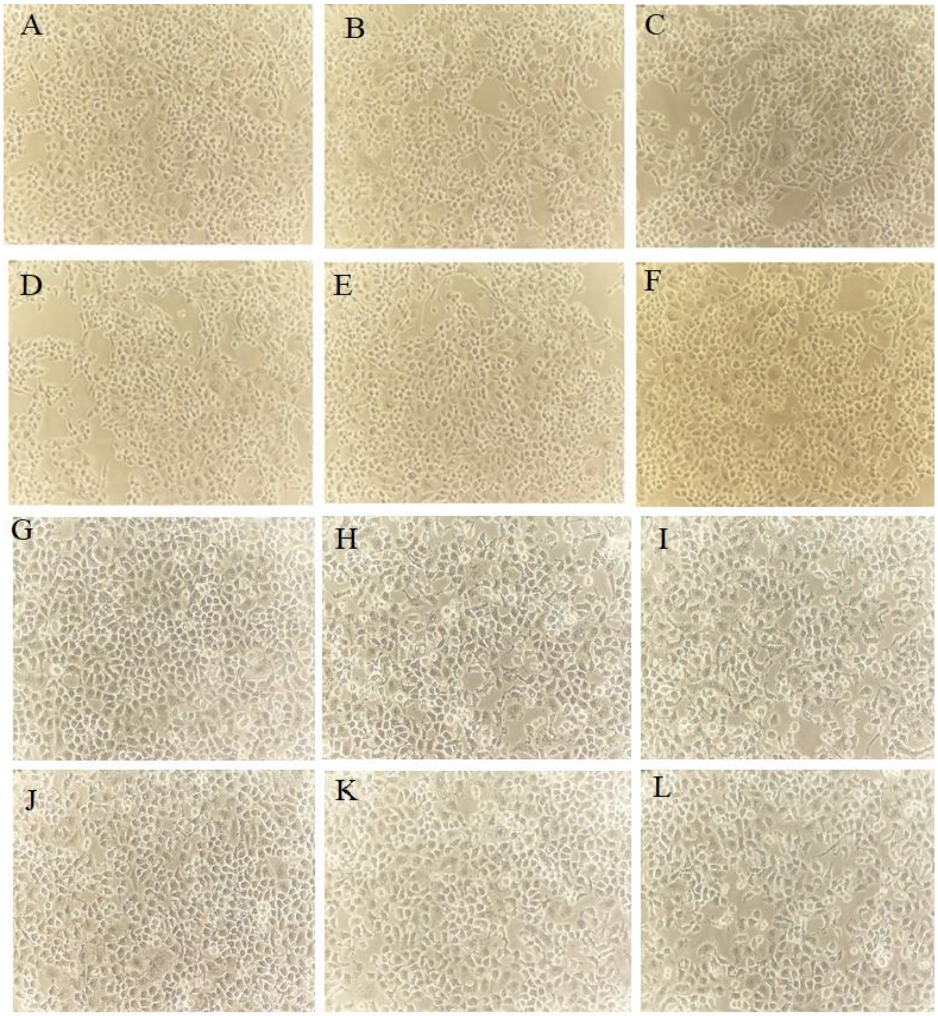

Figure S10. Microscopic images of EPG85.257-RDB cells after 72 h of incubation with 180 µg/ml of either NH_2-MSN (A), MSN-CS (B), MSN-CS-PEG-folate (C), MSN-CS-PEG-TAT (D), or MSN-CS-PEG-folate/TAT (E). EPG85.257-RDB cells without any treatments were considered as control (F). Microscopic images of HeLa-RDB cells after 72 h of incubation with 180 µg/ml of either NH_2-MSN (G), MSN-CS (H), MSN-CS-PEG-folate (I), MSN-CS-PEG-TAT (J), or MSN-CS-PEG-folate/TAT (K). HeLa-RDB cells without any treatments were considered as control (L). CS: chitosan, PEG: polyethylene glycol, TAT: trans-activator of transcription. Cells were pictured with 100X magnification.

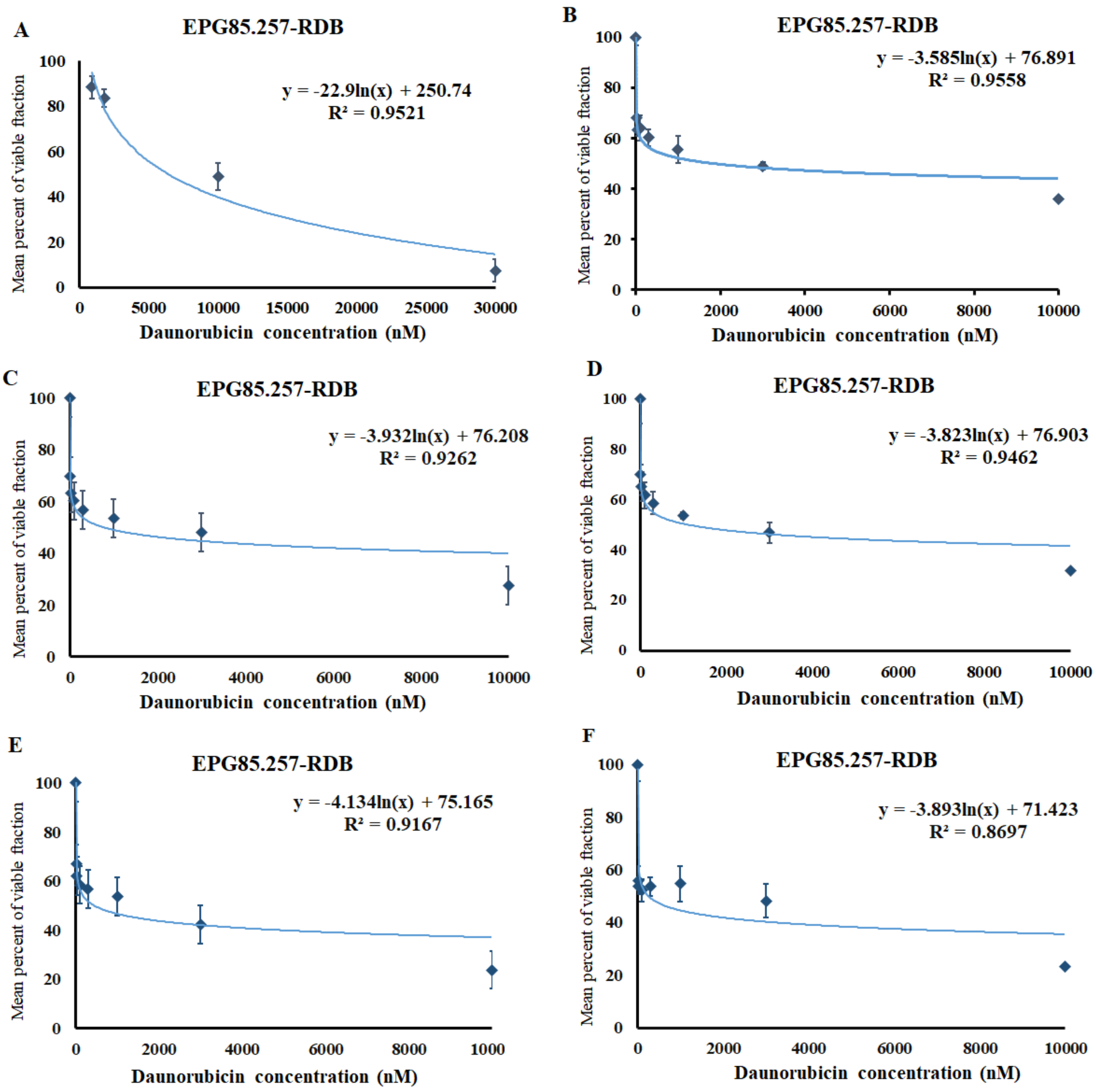

Figure S11. Daunorubicin cytotoxicity kinetics on EPG85.257-RDB in the absence (A) and presence (B-F) of different NH_2-MSN-siMDR1 nanostructures. NH_2-MSN-siRNA (B), NH_2-MSN-siRNA-CS (C), NH_2-MSN-siRNA-CS-folate (D), NH_2-MSN-siRNA-CS-TAT (E), NH_2-MSN-siRNA-CS-folate /TAT (F). CS: chitosan, TAT: trans-activator of transcription.

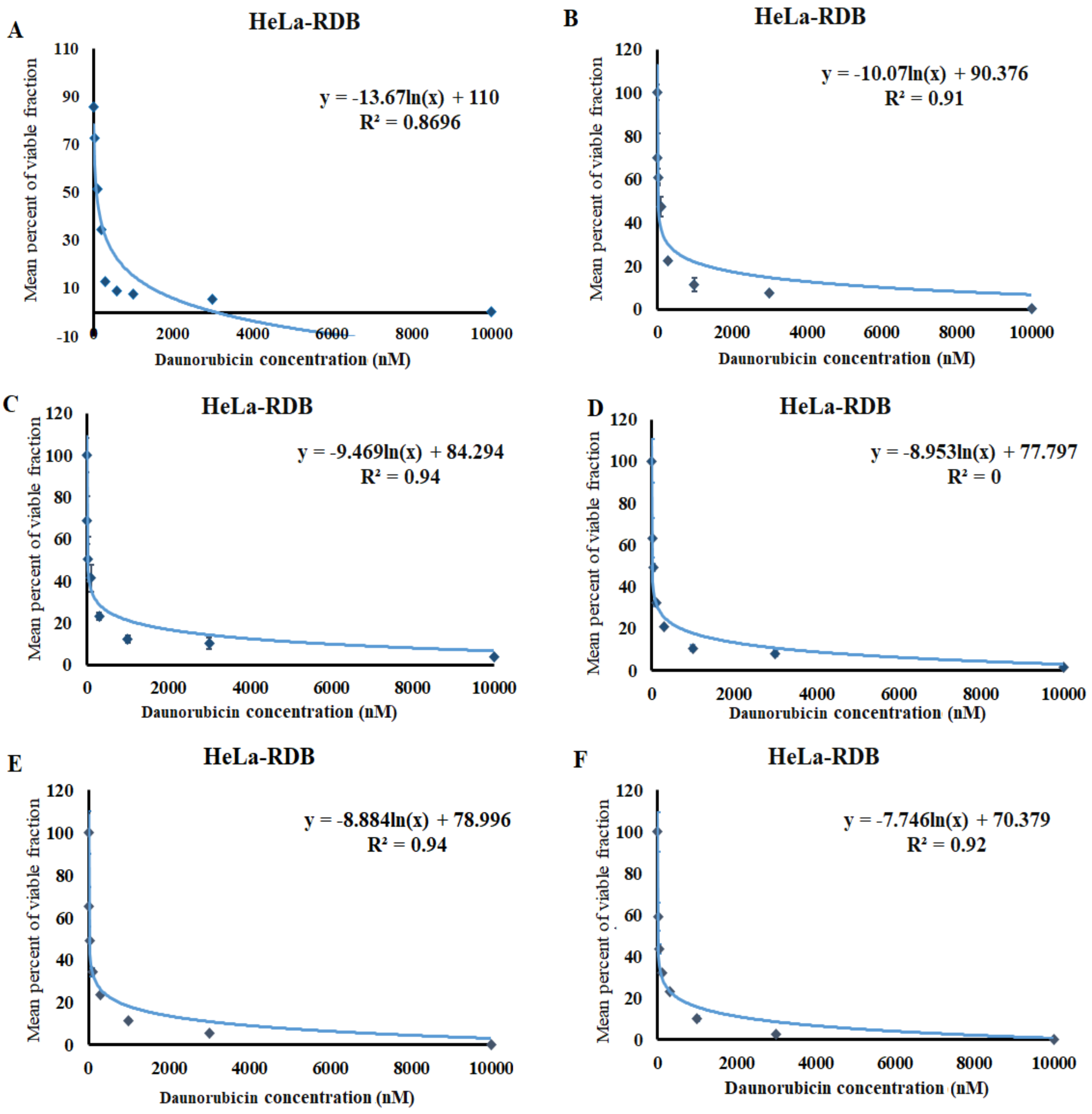

Figure S12. Daunorubicin cytotoxicity kinetic on HeLa-RDB in the absence (A) and presence (B-F) of different NH_2-MSN-siMDR1 nanostructures. NH_2-MSN-siRNA (B), NH_2-MSN-siRNA-CS (C), NH_2-MSN-siRNA-CS-folate (D), NH_2-MSN-siRNA-CS-TAT (E), NH_2-MSN-siRNA-CS-folate /TAT (F). CS: chitosan, TAT: trans-activator of transcription.

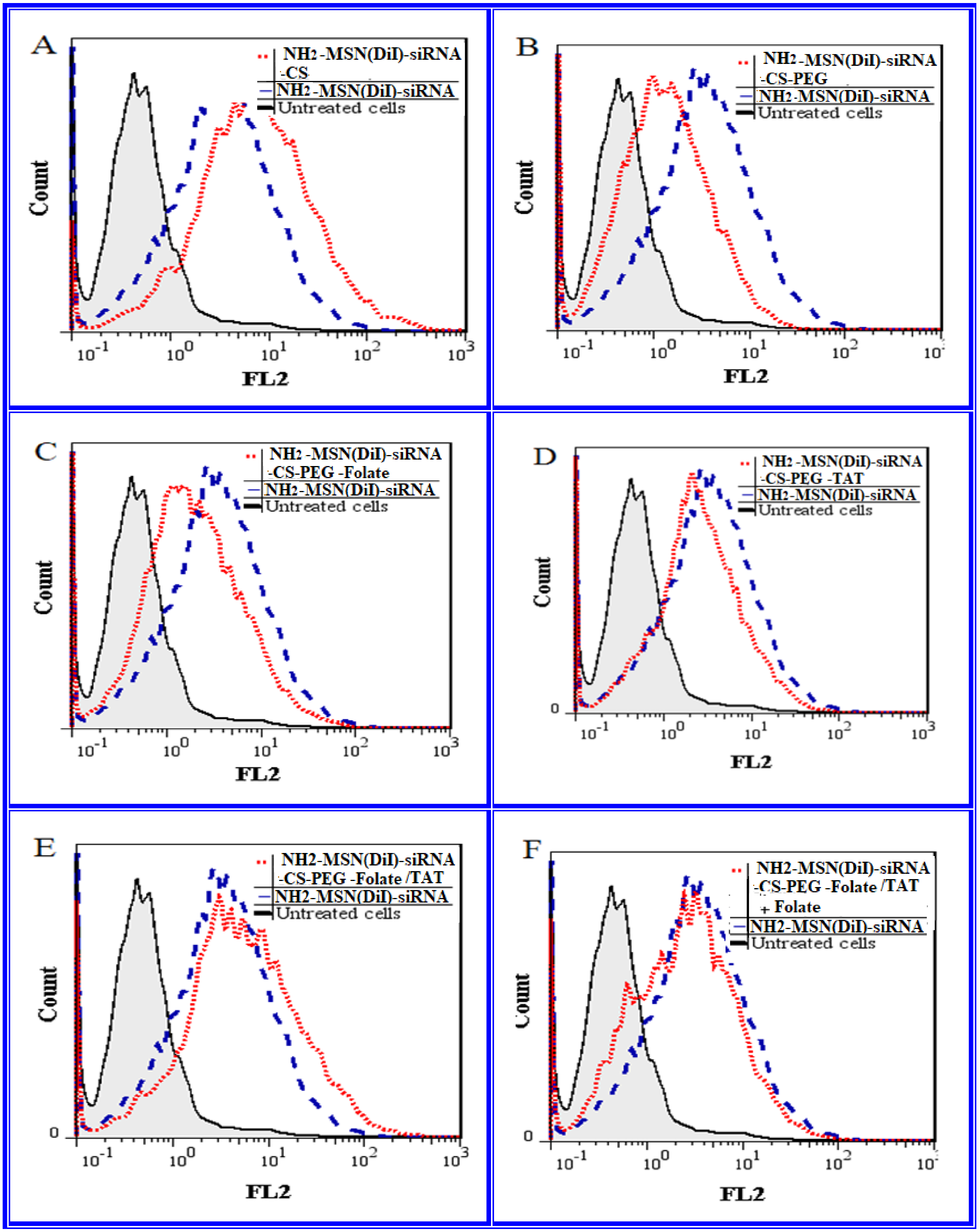
A
NH2-MSN(DiI)-siRNA -CS
NH2-MSN(DiI)-siRNA
Untreated cells
Count
FL2
B
NH2-MSN(DiI)-siRNA -CS-PEG
NH2-MSN(DiI)-siRNA
Untreated cells
C
NH2-MSN(DiI)-siRNA -CS-PEG-Folate
NH2-MSN(DiI)-siRNA
Untreated cells
D
NH2-MSN(DiI)-siRNA -CS-PEG-TAT
NH2-MSN(DiI)-siRNA
Untreated cells
E
NH2-MSN(DiI)-siRNA -CS-PEG-Folate/TAT
NH2-MSN(DiI)-siRNA
Untreated cells
F
NH2-MSN(DiI)-siRNA -CS-PEG-Folate/TAT + Folate
NH2-MSN(DiI)-siRNA
Untreated cells

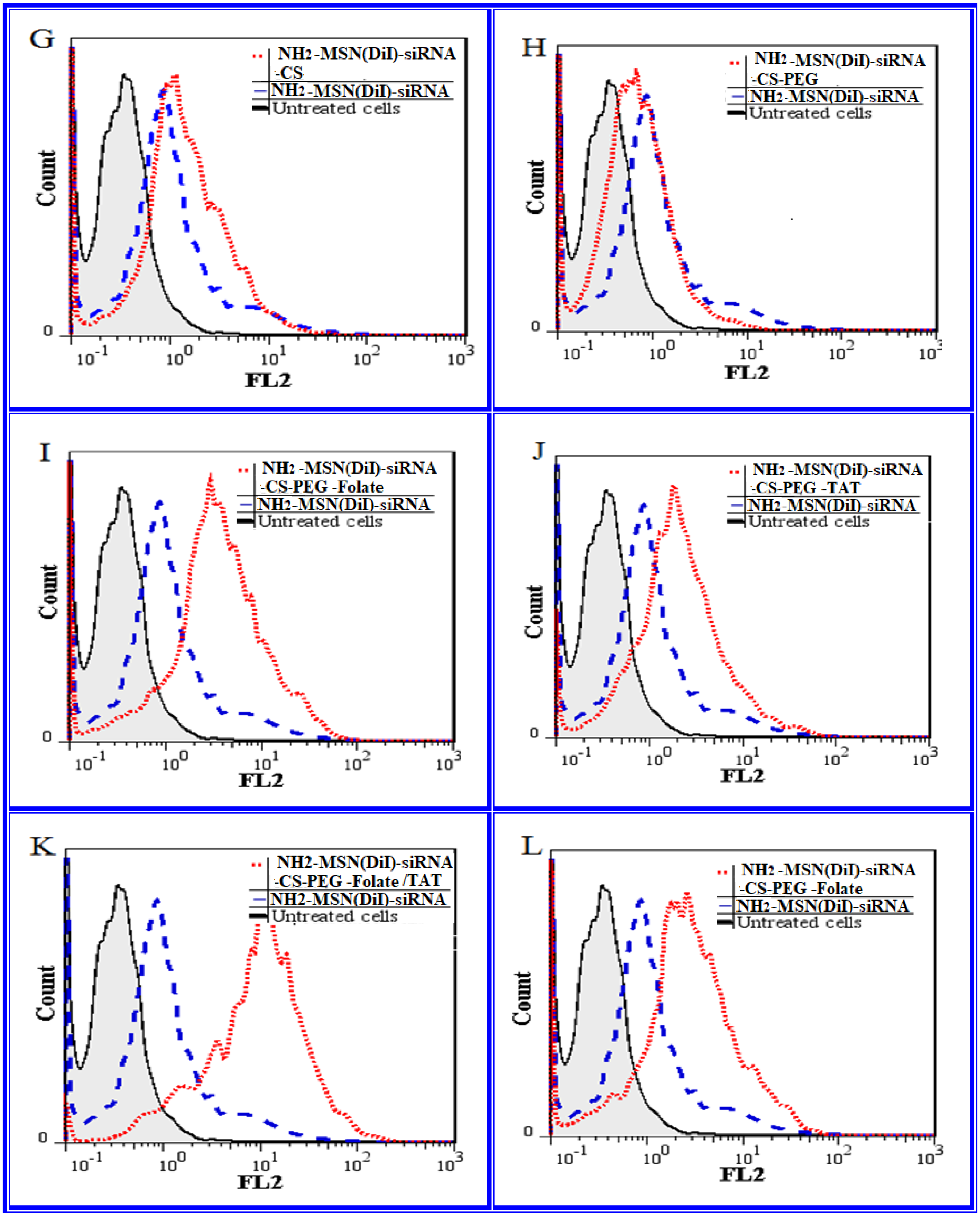
G
NH2-MSN(DiI)-siRNA -CS
NH2-MSN(DiI)-siRNA
Untreated cells
Count
0
10-1 100 101 102 103
FL2
H
NH2-MSN(DiI)-siRNA -CS-PEG
NH2-MSN(DiI)-siRNA
Untreated cells
Count
0
FL2
I
NH2-MSN(DiI)-siRNA -CS-PEG -Folate
NH2-MSN(DiI)-siRNA
Untreated cells
Count
0
10-1 100 101 102 103
FL2
J
NH2-MSN(DiI)-siRNA -CS-PEG -TAT
NH2-MSN(DiI)-siRNA
Untreated cells
Count
0
10-1 100 101 102 103
FL2
K
NH2-MSN(DiI)-siRNA -CS-PEG -Folate /TAT
NH2-MSN(DiI)-siRNA
Untreated cells
Count
0
10-1 100 101 102 103
FL2
L
NH2-MSN(DiI)-siRNA -CS-PEG -Folate
NH2-MSN(DiI)-siRNA
Untreated cells
Count
0
10-1 100 101 102 103
FL2

Figure S13. Flow cytometry histogram profile of the of EPG85.257-RDB (A-F) and HeLa-RDB (G-L) after 3 h of incubation with NH_2-MSN (DiI)-CS (A, G), NH_2-MSN (DiI)-CS-PEG (B, H), NH_2-MSN (DiI)-CS-PEG-folate (C, I), NH_2-MSN (DiI)-CS-PEG-TAT (D, J), NH_2-MSN (DiI)-CS-PEG-folate/TAT (E, K) and NH_2-MSN (DiI)-CS-PEG-folate/TAT+ excess free folate (F, L). Cells with NH_2-MSN (DiI) treatments were considered as control. The black histogram shows auto florescent of the cells (untreated cells). CS: chitosan, TAT: trans-activator of transcription.

Table S1. Primer Sequences and annealing temperature for Real-time PCR.

Genes	Primer Sequence	Annealing temperature	Accession number
**β-actin**	5'-TCATGAAGTGTGACGTGGACATC-3'<br>5'-CAGGAGGAGCAATGATCTTGATCT-3'	60 ºC	NM_001101.4
**MDR1**	5'-ATGAAGTTGAATTAGAAAATGCAG-3'<br>5'-GGAAACTGGAGGTATACTTTCATC-3'	56 ºC	NM_000927.4
**Folate receptor-α**	5'-AGGATTGCATGGGCCAGGACTG 3<br>5'-ATGGGCTTCCTGGCTGGTGTTG 3	60 ºC	NM_000802.3

(A)

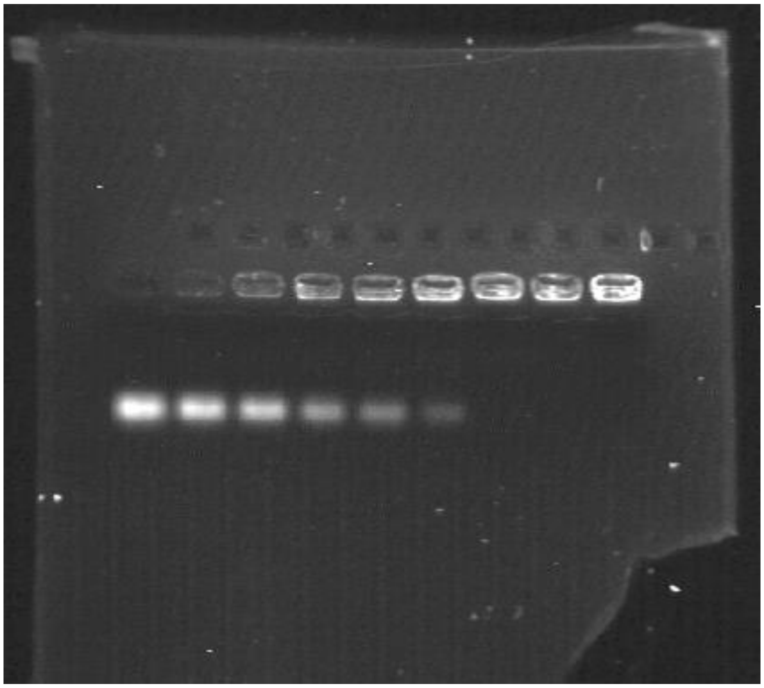

(B)

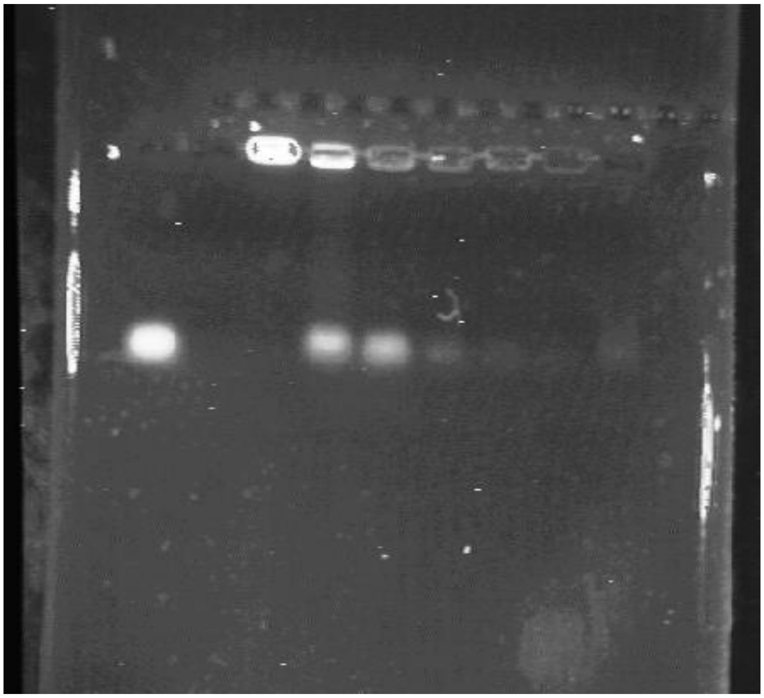

(C)

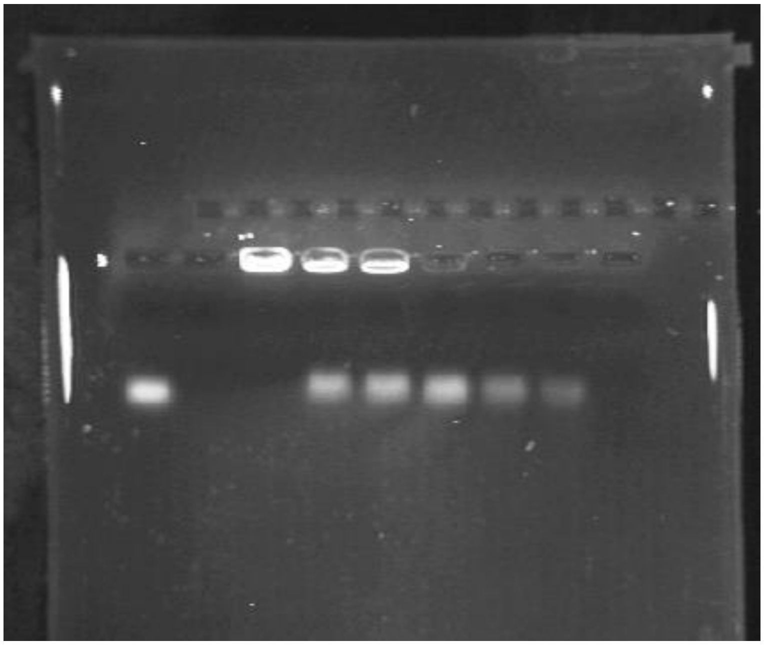

Figure S14. Original agarose gel electrophoresis pictures (represent figure 3 in the main text). Electrophoretic mobility pattern of siRNA/ NH_2-MSN complexes from left to right naked siRNA, 1:0.5, 1:1, 1:2, 1:3, 1:4, 1:5, 1:6, and 1:7 on agarose gel (A). RNase protection capacity for siRNA-loaded NH_2-MSNs (B) and siRNA-loaded NH_2-MSNs coated with chitosan (C). Wells from left to right are naked siRNA, naked siRNA treated with RNase, siRNA-loaded NH_2-MSNs, heparin treated NH_2-MSNs in the absence of the RNase, siRNA liberated from NH_2-MSNs using heparin in the presence of the RNase for 20 min, for 40 min, for 60 min, and finally 120 min. Gel analysis revealed that chitosan coating on the NH_2-MSNs could provide better safety for the loaded siRNA than NH_2-MSNs without chitosan.

First western blotting round

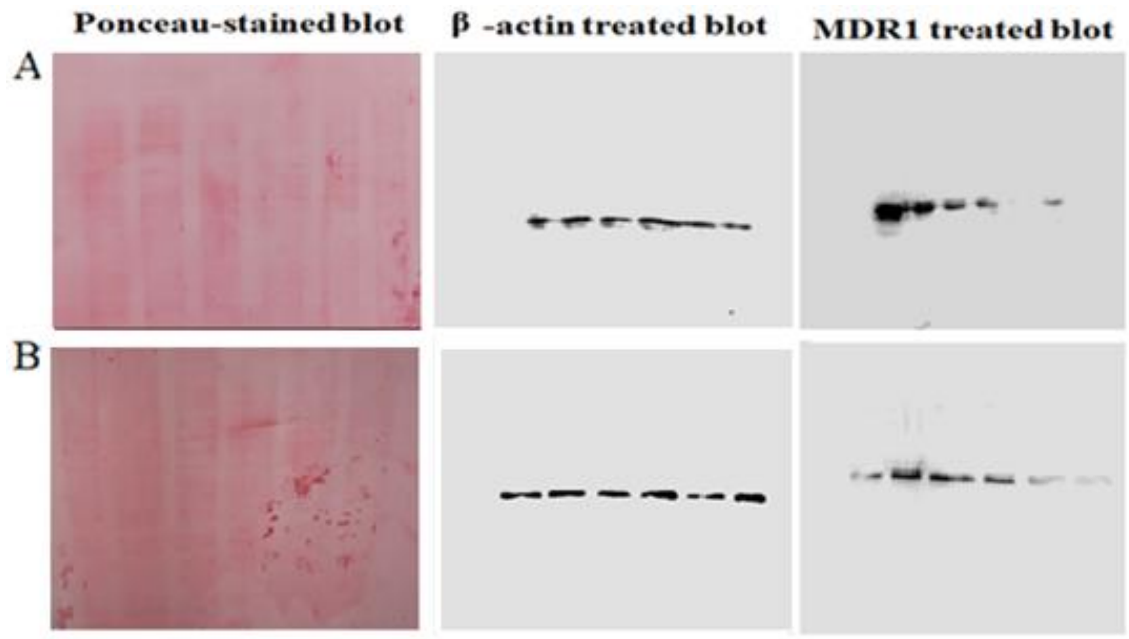

Second western blotting round

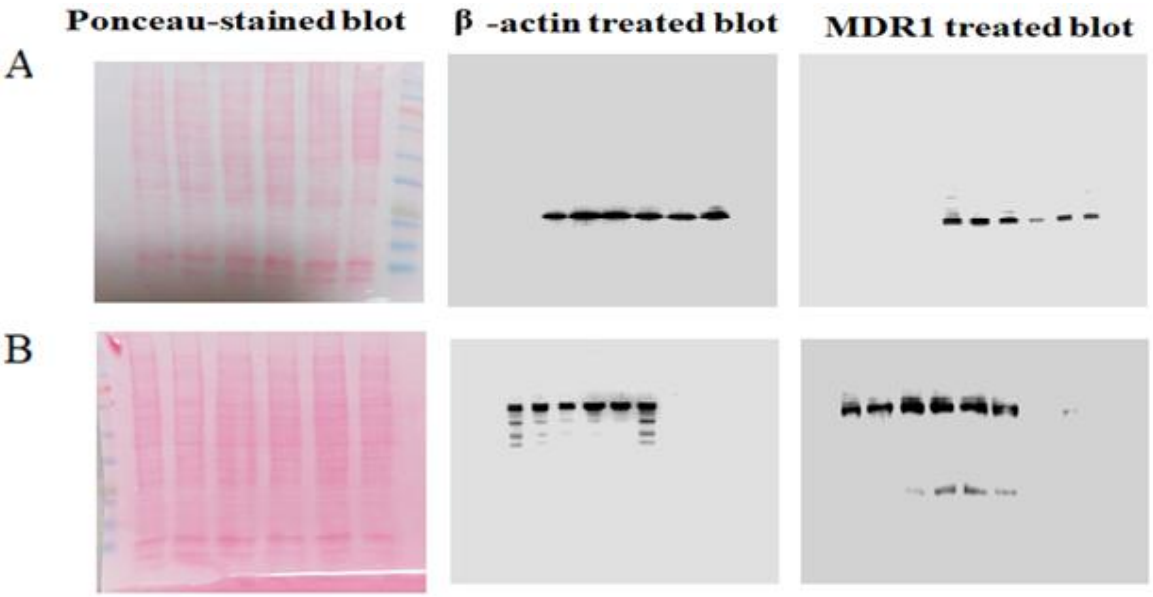

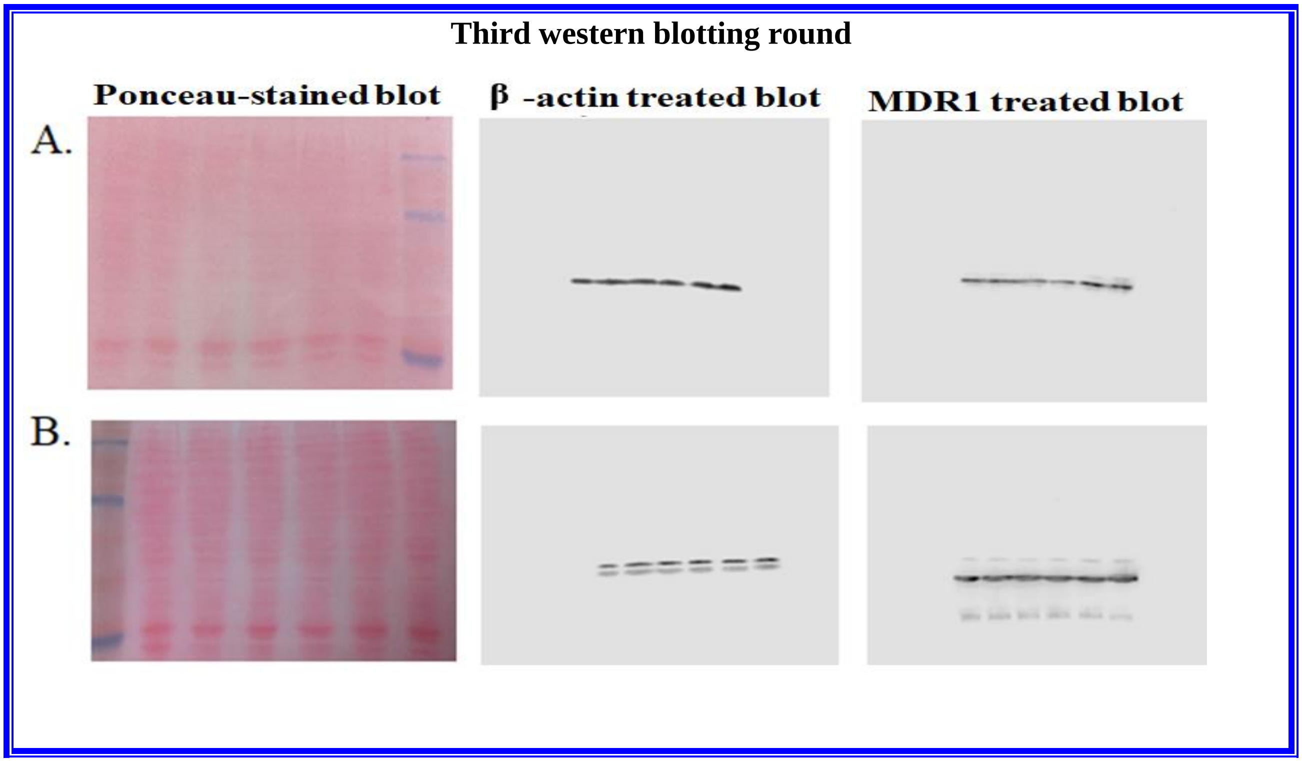

Figure S15. Original western blot pictures of HeLa-RDB (A) and EPG85.257-RDB (B) (represent figure 7 in the main text). MDR1 gene knockdown were evaluated by western of MDR1 protein in cells in presence NH_2-MSN-siRNA, NH_2-MSN-siRNA-CS, NH_2-MSN-siRNA-CS-folate, NH_2-MSN-siRNA-CS-TAT and NH_2-MSN-siRNA-CS-folate/TAT. Briefly, whole protein extracts were prepared at each individual time point and subjected to western blotting. 50 μg protein was electrophoresed on 12% SDS-PAGE. Proteins were transferred to nitrocellulose membranes. Then blots were incubated overnight at 4 ^{o}C with primary antibodies of MDR1 (Santa Cruz Biotechnology, sc-55510) and then incubated with mouse IgG kappa binding protein conjugated to horseradish peroxidase secondary antibody (m-IgGκ BP-HRP, Santa Cruz Biotechnology, sc-516102) for 2 h at room temperature. β-Actin (Santa Cruz Biotechnology, sc-47778) was used as an internal standard protein. A predesigned Human ABCB1 siRNA: Cat Number: 5243-1, 5243-2, 5243-3 (20 nmole/BioRP) and AccuTarget™ Negative Control siRNA (Cat Number: SN-1012, 10 nmole/BioRP) both from Bioneer company were used in the experiment. Negative control is NC-siRNA loaded NH_2-MSN-chitosan-PEG-folate/TAT construct. Each round is an independent cell experiment. CS: chitosan, TAT: trans-activator of transcription.
